# The caveolae‐associated coiled‐coil protein, NECC2, regulates insulin signalling in Adipocytes

**DOI:** 10.1111/jcmm.13840

**Published:** 2018-08-30

**Authors:** Andrés Trávez, Yoana Rabanal‐Ruiz, Jaime López‐Alcalá, Laura Molero‐Murillo, Alberto Díaz‐Ruiz, Rocío Guzmán‐Ruiz, Victoria Catalán, Amaia Rodríguez, Gema Frühbeck, Francisco J. Tinahones, Stéphane Gasman, Nicolas Vitale, Yolanda Jiménez‐Gómez, María M. Malagón

**Affiliations:** ^1^ Instituto Maimónides de Investigación Biomédica de Córdoba (IMIBIC) Córdoba Spain; ^2^ Department of Cell Biology, Physiology, and Immunology University of Córdoba Córdoba Spain; ^3^ Reina Sofía University Hospital Córdoba Spain; ^4^ CIBER Fisiopatología de la Obesidad y Nutrición (CIBERobn) Instituto de Salud Carlos III Madrid Spain; ^5^ Metabolic Research Laboratory Department of Endocrinology & Nutrition Clínica Universidad de Navarra IdiSNA Pamplona Spain; ^6^ Unidad de Gestion Clínica de Endocrinología y Nutrición Laboratorio del Instituto de Investigación Biomédica de Málaga (IBIMA) Hospital Universitario de Málaga (Virgen de la Victoria) Universidad de Málaga Málaga Spain; ^7^ Institut des Neurosciences Cellulaires et Intégratives (INCI) Centre National de la Recherche Scientifique (CNRS UPR 3212) Université de Strasbourg Strasbourg France

**Keywords:** adipocytes, adipogenesis, caveolae, insulin resistance, insulin signalling, NECC2, obesity

## Abstract

Adipocyte dysfunction in obesity is commonly associated with impaired insulin signalling in adipocytes and insulin resistance. Insulin signalling has been associated with caveolae, which are coated by large complexes of caveolin and cavin proteins, along with proteins with membrane‐binding and remodelling properties. Here, we analysed the regulation and function of a component of caveolae involved in growth factor signalling in neuroendocrine cells, neuroendocrine long coiled‐coil protein‐2 (NECC2), in adipocytes. Studies in 3T3‐L1 cells showed that NECC2 expression increased during adipogenesis. Furthermore, NECC2 co‐immunoprecipitated with caveolin‐1 (CAV1) and exhibited a distribution pattern similar to that of the components of adipocyte caveolae, CAV1, Cavin1, the insulin receptor and cortical actin. Interestingly, NECC2 overexpression enhanced insulin‐activated Akt phosphorylation, whereas NECC2 downregulation impaired insulin‐induced phosphorylation of Akt and ERK2. Finally, an up‐regulation of *NECC2* in subcutaneous and omental adipose tissue was found in association with human obesity and insulin resistance. This effect was also observed in 3T3‐L1 adipocytes exposed to hyperglycaemia/hyperinsulinemia. Overall, the present study identifies NECC2 as a component of adipocyte caveolae that is regulated in response to obesity and associated metabolic complications, and supports the contribution of this protein as a molecular scaffold modulating insulin signal transduction at these membrane microdomains.

## 1 INTRODUCTION

Type 2 diabetes (T2D) is characterized by the progressive deterioration of glycaemic control. The first recognizable abnormality detected in individuals destined to develop T2D is insulin resistance, in which insulin action is impaired in skeletal muscle, liver and adipose tissue.^1^, ^2^ Insulin exerts its physiological actions upon binding to the insulin receptor (IR), leading to the activation of two major signalling pathways: the phosphatidylinositol 3‐kinase (PI3K)‐protein kinase B (PKB)/Akt pathway, which is responsible for the metabolic actions of insulin, and the Ras‐MAPK pathway, which regulates gene expression and interacts with the PI3K pathway to control cell growth and differentiation.^3^, ^4^ Despite an ever‐growing list of molecules that appear to be required for insulin signalling, numerous gaps remain in our understanding of the precise molecular mechanisms that control these signal transduction pathways.

Caveolae are plasma membrane microdomains enriched in cholesterol and sphingolipids that act as signalling platforms containing a variety of signalling receptors and enzymes.^5^, ^6^ They have been also involved in endocytosis, cholesterol homeostasis, apoptosis and proliferation.^7^, ^8^, ^9^, ^10^ On their cytoplasmic side, caveolae are coated with large complexes of caveolin and cavin proteins, along with several other proteins with membrane‐binding and ‐remodelling properties such as dynamin, the dynamin‐like ATPase EHD2 or the BAR‐domain protein‐containing protein PACSIN2.^9^, ^11^ Caveolae are particularly abundant in adipocytes.^12^ Evidence suggesting that caveolae and caveolins may play a role in IR signalling came from experiments demonstrating that the scaffolding domain of caveolin‐1 (CAV1) binds to a small specific motif in the tyrosine kinase domain of the IR essential for tyrosine kinase activity (residues 1193‐1200).^13^ Besides caveolins, other proteins with scaffolding properties may represent additional relevant organizers of signal transduction at the caveolae.^14^ Nevertheless, the molecular components of the caveolae‐associated insulin signalling system remain to be fully elucidated.

Long alpha‐helical coiled‐coil proteins represent highly versatile molecules that have been proposed to act as molecular scaffolds and/or tethers that stabilize and organize membrane systems.^15^ Furthermore, these proteins have been suggested to integrate signals and transduction pathways through their ability to interact with multiple signalling components.^15^ We recently identified a long coiled‐coil protein, referred to as neuroendocrine long coiled‐coil protein‐2 (NECC2),^16^, ^17^ which associates with caveolae in neuroendocrine cells, wherein it colocalizes with the nerve growth factor (NGF) receptor, TrkA, and regulates TrkA‐mediated NGF signalling.^18^ Remarkably, the presence and function of NECC2 in caveolae in adipocytes has not been yet documented.

The aim of the present study was to investigate the regulation and function of NECC2 in adipocytes and to establish its relationship to obesity and insulin sensitivity. Confocal microscopy studies showed that NECC2 immunosignal colocalized with that of CAV1, Cavin1, β‐actin and IR at the cell surface of 3T3‐L1 adipocytes. We also show that overexpression of NECC2, which co‐immunoprecipitated with CAV1, enhanced insulin‐activated Akt phosphorylation, whereas NECC2 silencing impaired IR‐dependent activation of extracellular‐regulated kinase 2 (ERK2) and Akt. Moreover, we observed that *NECC2* expression in human omental and subcutaneous adipose tissue increased in obesity and, in particular, in relation to insulin resistance. Furthermore, *in vitro* induction of insulin resistance by chronic exposure of 3T3‐L1 adipocytes to high concentrations of glucose and insulin also increased NECC2 content. Taken together, our data indicate that NECC2 is a component of adipocyte caveolae that is regulated in response to obesity and associated metabolic complications, and support a role for this protein as a molecular scaffold modulating insulin signal transduction at these membrane microdomains.

## 2 MATERIALS AND METHODS

### 2.1 Antibodies and reagents

A polyclonal rabbit antiserum against rat NECC2 (residues 2‐17), anti‐NECC2, was produced and affinity‐purified as described.^18^ All other antibodies and dilutions employed are shown in Table S1. Phalloidin was from Invitrogen (Carlsbad, CA, USA) and latrunculin B from Calbiochem (Darmastadt, Germany). Unless otherwise indicated, all other reagents were purchased from Sigma‐Aldrich (Madrid, Spain).

### 2.2 Cell culture and *in vitro* experimental setups

3T3‐L1 cells (ATCC; Manassas, VA, USA) were differentiated into adipocytes.^19^ NECC2 expression and protein content was assessed at days 0, 3, 6, 10 and 12 of differentiation. For experimental treatments, 3T3‐L1 adipocytes at day 8‐10 of differentiation were preincubated in serum‐free culture medium (2 hours) and then cultured in the absence or presence of the following test substances: insulin (100 nmol/L, up to 40 minutes), latrunculin B (5 μmol/L, 30 minutes), methyl‐β‐cyclodextrin (βMCD; 10 mmol/L, 90 minutes), palmitate (500 μmol/L, 18 hours), oleate (500 μmol/L, 18 hours), TNF‐α (5 nmol/L, 24 hours) or a combination of high glucose (25 nmol/L) and high insulin (100 nmol/L) (HGHI) for 24 hours.

At the end of the experiments, cells were harvested for RNA and/or protein determination or processed for confocal microscopy.

### 2.3 Human studies

Samples of omental and subcutaneous adipose tissue were obtained from the abdominal region of 45 Caucasian individuals (22 males, 23 females) undergoing diverse laparoscopic surgery procedures after ethics committee approval was obtained at the Clínica Universidad de Navarra (Pamplona, Spain). The study was conducted according to the principles of the Declaration of Helsinki. All participants provided written informed consent.

Patients underwent a clinical assessment including medical history, physical examination and body composition analysis (Table S2). Obese subjects (≥30 kg/m^2^) were sub‐classified into three groups [normoglycemic (NG), impaired glucose tolerance (IGT) or T2D] following the criteria of the Expert Committee on the Diagnosis and Classification of Diabetes.^20^ T2D subjects were not on insulin therapy or on medication likely to influence endogenous insulin levels. Biochemical and hormonal assays were carried out as previously described.^21^

Tissue samples were immediately frozen in liquid nitrogen and stored at −80°C until use.

### 2.4 RNA isolation and expression analysis by RT‐PCR

Total RNA from 3T3‐L1 cells was extracted using the TRIzol method (Tri^®^ Reagent) following the manufacturer′s instructions.^19^ RNA isolation and purification from human adipose tissue samples were performed as described.^22^ The expression levels of *NECC2* gene, and of *18s* ribosomal RNA (rRNA) as a housekeeping gene, were measured by real‐time PCR using an iCycler™ Real‐Time PCR System (Bio‐Rad Laboratories, Hercules, CA, USA). Primers are listed in Table S3. For cDNA quantification, a standard curve‐based method for relative real‐time PCR data processing was used. All measurements were performed in duplicate and the average values were calculated. Controls consisting of reaction mixture without cDNA were negative in all runs.

### 2.5 Immunocytochemistry

3T3‐L1 adipocytes were fixed in 4% w/v paraformaldehyde (15 minutes), incubated with PBS containing 0.3% w/v saponin and 1% w/v BSA (1 hours at RT), and then exposed (overnight, 4°C) to rabbit anti‐NECC2 antibody,^18^ alone or in combination with antibodies against CAV1, Perilipin1, Cavin1 or IR (Table S1). After incubation, an Alexa594‐conjugated secondary antibody alone or in combination with an Alexa488‐conjugated secondary antibody was added. Actin filaments were visualized by phalloidin staining (0.15 μmol/L, 30 minutes). Samples were visualized under a TCS‐SP2‐AOBS (Leica Corp., Heidelberg, Germany) or ZEISS LSM700 (Carl Zeiss AG., Oberkochen, Germany) confocal laser scanning microscope. Confocal images were processed using the Huygens Essential software package (SVI, Hilversum, The Netherlands). The degree of colocalization of the fluorescence signals was estimated by determining an overlapping pixel map of the channels (ie, a mask) using the Colocalization Finder plugin for ImageJ (NIH, Bethesda, MA, USA) and Manders′ coefficient (MC) using Imaris software (Bitplane, Zurich, Switzerland). Negative controls without primary antibodies were included to assess non‐specific staining.

### 2.6 Immunoprecipitation assay

A standard protocol was used for co‐immunoprecipitation of NECC2 and CAV1 in HEK‐293 AD cells transfected with c‐Myc‐*Necc2* and CFP‐*Cav1* as described previously.^23^

### 2.7 Subcellular fractionation studies

Post‐nuclear supernatant (PNS), cytosolic (S2) and crude membrane (P2) fractions of 3T3‐L1 adipocytes at baseline and after 30 minutes of insulin treatment (100 nmol/L) were obtained by subcellular fractionation.^18^ Protein distribution was analysed by immunoblotting.

Caveolin‐enriched membrane fractions were isolated from 3T3‐L1 adipocytes using a detergent‐free method and sucrose gradient fractionation analysis as previously described.^18^ After ultracentrifugation, 9 fractions (450 μL) were collected from the top of the sucrose gradient and analysed by SDS‐PAGE.

### 2.8 Immunoblotting

Protein extracts were obtained from cells lysed in buffer containing 50 mmol/L Tris‐HCl (pH 7.40), 150 mmol/L NaCl, 1% v/v Triton‐X‐100, 1 mmol/L EDTA and 1 μg/mL anti‐protease cocktail. 3T3‐L1 adipocytes from overexpression and silencing studies were lysed in SDS‐DTT.^24^

Protein extracts were separated by SDS‐PAGE. Antibodies against NECC2, CAV1, IR, Adiponectin, Cavin1, Perilipin1, B‐actin, A‐tubulin, Akt, pAkt, ERK1/2, p44/42 MAPK, c‐Myc and GFP were dispensed overnight (4°C) and peroxidase‐conjugated secondary antibodies were administered for 1 hour (RT). The immunoreaction was visualized using ECL plus (GE Healthcare, Buckinghamshire, UK). B‐actin, A‐tubulin and Ponceau S^25^, ^26^ were selected as loading controls. Densitometric analysis of the immunoreactive bands was carried out with Image J software.

### 2.9 Overexpression and silencing studies

3T3‐L1 adipocytes were electroporated (Gene PulserXcell, Bio‐Rad) as previously described.^19^ For overexpression analysis, cells were electroporated with a phrGFP‐N1 expression vector (mock‐transfected cells) or a GFP‐*Necc2* construct 18 and cultured for 48 hours prior to the experiments. For silencing studies, a specific shRNA for *Necc2* silencing (5′‐GGAGGAGATAAGATTTAAA‐3′) was cloned using the BgIII and HindIII sites in front of the H1‐RNA promoter of the pEGFP‐RNAi plasmid as described earlier.^18^ Cells expressing pEGFP‐shRNA (control shRNA) or pEGFP‐*Necc2*‐shRNA plasmid (NECC2 shRNA) were kept in culture for 72 hours before performing the experiments.

For colocalization analysis of NECC2 and cavin1, cells were transfected with Lipofectamine 2000 (Invitrogene, Barcelona, Spain) and the expression vector coding for GFP‐*Necc2*, cultured for 48 hours and then immunostained for cavin1. For Cav1 silencing studies, 3T3‐L1 adipocytes were transfected with Lipofetamine RNAiMAX (Invitrogen) and *Cav1* siRNA (Dharmacon, Lafayette, CO, USA) and cultured for 72 hours before NECC2 immunostaining.

### 2.10 Statistical analysis

Statistical analysis was carried out using SPSS statistical software, version 19.0 for WINDOWS (SPSS Inc., Chicago, IL, USA). The normal distribution of variables was assessed using the Kolmogorov‐Smirnov test and log‐transformed if appropriate. Repeated measures ANOVA (RM‐ANOVA), One‐Way ANOVA, Independent‐Samples *t* test and paired‐samples *t* test were used where appropriate. A Bonferroni correction was applied for multiple testing. A post‐hoc statistical analysis using the Tukey or Games‐Howell′s test was used to identify significant differences between groups. The contrast statistic used when the sphericity assumption was not satisfied was Huynh‐Feldt. Values were considered significant at *P *<* *0.05.

## 3 RESULTS

### 3.1 NECC2 expression increases during differentiation of 3T3‐L1 cells

**Figure 1:**
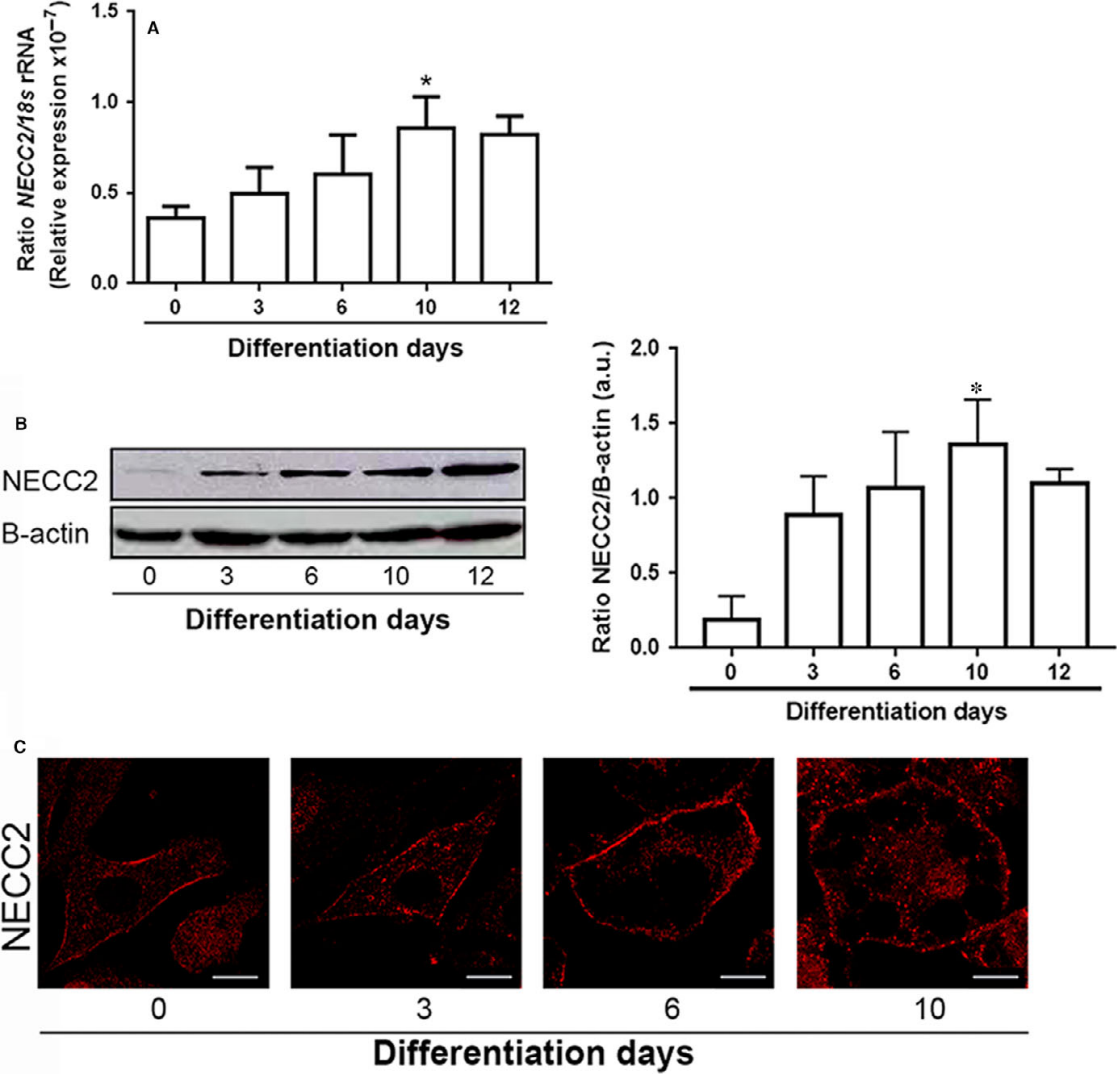
NECC2 expression and distribution during differentiation of 3T3‐L1 cells into adipocytes. A, Quantitative RT‐PCR analysis of *Necc2* mRNA levels in 3T3‐L1 cells exposed to a hormonal differentiation cocktail for 0, 3, 6, 10 and 12 d. Gene expression was represented as ratio of target gene concentration to the concentration of a housekeeping gene, the *18s* rRNA. Data represent the mean ± SEM of four independent experiments. Data were analysed for significance using paired‐samples *t* test. *P <* *0.05 *vs* 0 d. B, Representative immunoblot of NECC2 protein content in 3T3‐L1 cell extracts during differentiation. B‐actin immunosignal was used as reference for protein charge. The graph shows the mean ± SEM from 5 independent experiments. Data were analysed for significance using paired‐samples *t* test. *P <* *0.05 *vs* 0 d. C, Representative confocal images of 3T3‐L1 cells immunolabeled for NECC2 during differentiation. Scale bar 10 μm

We quantified *Necc2* mRNA content in 3T3‐L1 cells at 0, 3, 6, 10 and 12 days of differentiation by RT‐PCR. *Necc2* expression increased throughout adipogenesis, reaching a peak at day 10 (*P *=* *0.041) (Figure 1A). Adiponectin and Oil Red O were used as controls of adipogenesis (Figure S1a).

Immunoblot analysis of NECC2 using a polyclonal antibody raised against the N‐terminal of NECC2 (anti‐NECC2) 18 revealed an immunoreactive band of approximately 110 kg/mol in protein extracts from 3T3‐L1 adipocytes. Immunoreaction was abolished after preadsorption of the antibody with the purified antigen and in *Necc2* shRNA transfected cells (Figure S1b). NECC2 protein content also increased throughout differentiation, reaching a peak at day 10 (*P* = 0.045) (Figure 1B). Immunostaining of 3T3‐L1 cells with anti‐NECC2 antibody revealed that NECC2 immunosignal was distributed diffusely throughout the cytoplasm and in close apposition to the plasma membrane, both in undifferentiated and differentiated 3T3‐L1 adipocytes (Figure 1C). NECC2 immunolabeling was significantly reduced in cells expressing *Necc2* shRNA (Figure S1c).

### 3.2 NECC2 is localized to caveolae in 3T3‐L1 adipocytes

**Figure 2:**
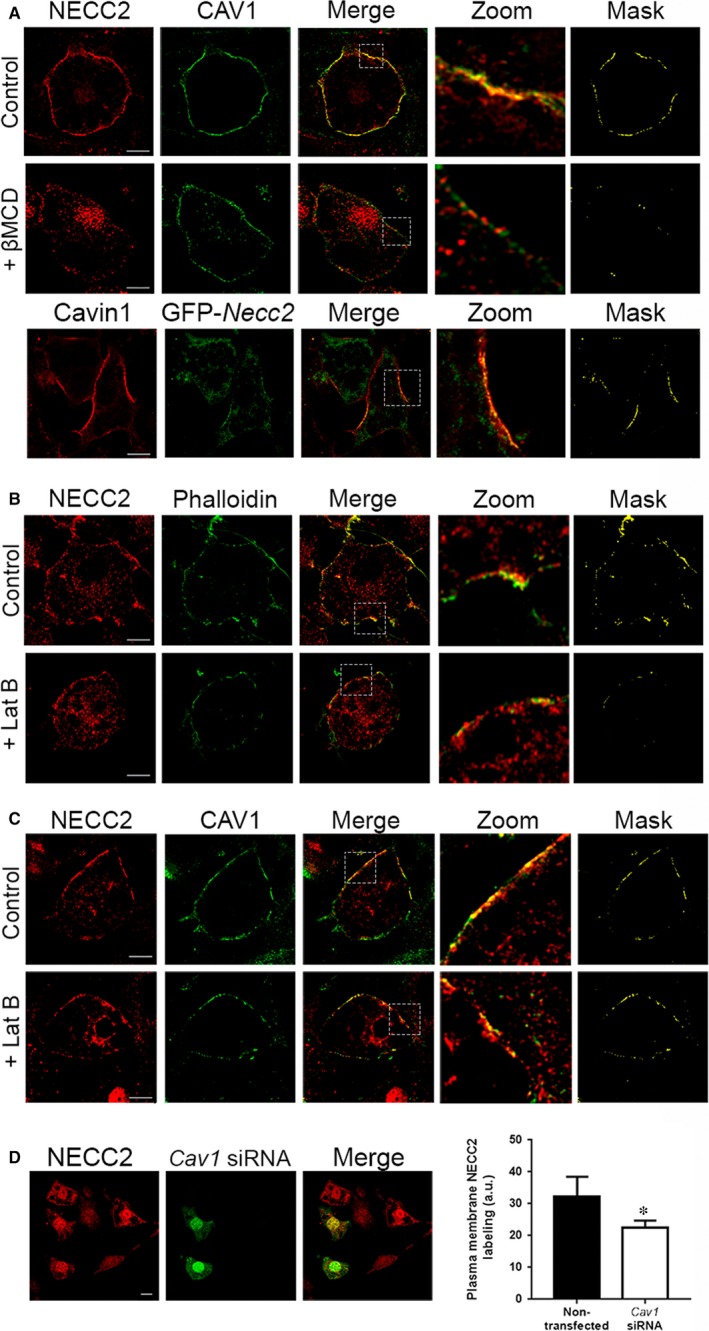
Subcellular localization of NECC2 in 3T3‐L1 adipocytes. (A) Confocal microscope images of 3T3‐L1 adipocytes under basal conditions (top panels) or challenged with 10 mmol/L methyl‐β‐cyclodextrin (βMCD) (middle panels) for 90 min, and co‐immunostained for NECC2 (red) and the caveolae marker caveolin‐1 (CAV1) (green). Bottom panels represent confocal microscope images of 3T3‐L1 adipocytes expressing GFP‐*Necc2* (green) and immunostained for Cavin1 (red). Colocalization of the two immunosignals can be observed in the two most right panels [magnified insets (zoom) and binary mask]. Scale bar 10 μm (B) Confocal microscope images of 3T3‐L1 adipocytes under basal conditions (top panels) or challenged with 5 μmol/L latrunculin B (LatB) (bottom panels) for 30 min, and double‐stained with NECC2 (red) and actin marker phalloidin (green). Significant overlap between markers at the cell periphery is shown in the magnified insets (zoom) and binary mask at the most right panels. Scale bar 10 μm (C) Confocal microscope images of 3T3‐L1 adipocytes under basal conditions (top panels) or challenged with 5 μmol/L latrunculin B (LatB) (bottom panels) for 30 min, and co‐immunostained for NECC2 (red) and the caveolae marker CAV1 (green). Colocalization of the two immunosignals can be observed in the two most right panels [magnified insets (zoom) and binary masks]. Scale bar 10 μm. (D) Cell surface NECC2 labelling after *Cav1* silencing. Differentiated 3T3‐L1 cells transiently transfected with *Cav1* siRNA were double‐stained for NECC2 (red) and *Cav1* siRNA (green). Scale bar 10 μm. The graph represents the quantification of NECC2 immunosignal at the membrane level, and the results are expressed as mean ± SEM of at least 10 cells per experimental group (n = 20)

Previous studies demonstrated the colocalization of NECC2 with CAV1 in caveolae in neuroendocrine PC12 cells.^18^ Likewise, examination of double immunostained 3T3‐L1 adipocytes by confocal microscopy showed a high degree of overlap between NECC2 and CAV1 immunosignals, as well as between those of NECC2 and Cavin1 at the cell surface (MC = 0.1168 ± 0.011 (n = 10) and MC = 0.7407 ± 0.0796 (n = 6), respectively) (Figure 2A). No colocalization was found between NECC2 and the lipid droplet‐associated protein, Perilipin1 (Figure S3a). The localization of NECC2 to caveolae was confirmed after disruption of these membrane domains by cholesterol depletion with methyl‐β‐cyclodextrin. This treatment reduced the intensity of both NECC2 and CAV1 immunosignals at the plasma membrane, leading to a loss of colocalization between the two markers (MC = 0.1277 ± 0.1549 *vs* 0.0913 ± 0.1805 (n = 10), *P *=* *0.006) (Figure 2A).

We also explored the colocalization of NECC2 and actin given the association of this cytoskeletal component with caveolae in adipocytes.^27^ This showed that NECC2 immunosignal partially overlaped with phalloidin at the cell cortex (MC = 0.2026 ± 0.00014, n = 10) (Figure 2B). Pretreatment of 3T3‐L1 adipocytes with latrunculin B, an F‐actin dissembler, reduced the overlapping degree for NECC2 and cortical actin at the plasma membrane (MC=0.2026 ± 0.00014 *vs* 0.0637 ± 0.0084 (n = 10), *P *=* *0.039), without modifying NECC2 distribution at the plasma membrane (Figure 2B). Indeed, latrunculin B‐induced actin depolymerization in 3T3‐L1 adipocytes did not modify the colocalization index for NECC2 and CAV1 at the cell surface (MC = 0.1004 ± 0.0098 *vs* 0.1240 ± 0.004 (n = 10), *P *=* *0.153) (Figure 2C). Additionally, 3T3‐L1 adipocytes were transfected with a *Cav1* siRNA, which reduced CAV1 content by 88% but had no effect on NECC2 content (Figure S3b). However, down‐regulation of CAV1 expression reduced by 30% NECC2 immunosignal intensity at the adipocyte surface (Figure 2D).

**Figure 3:**
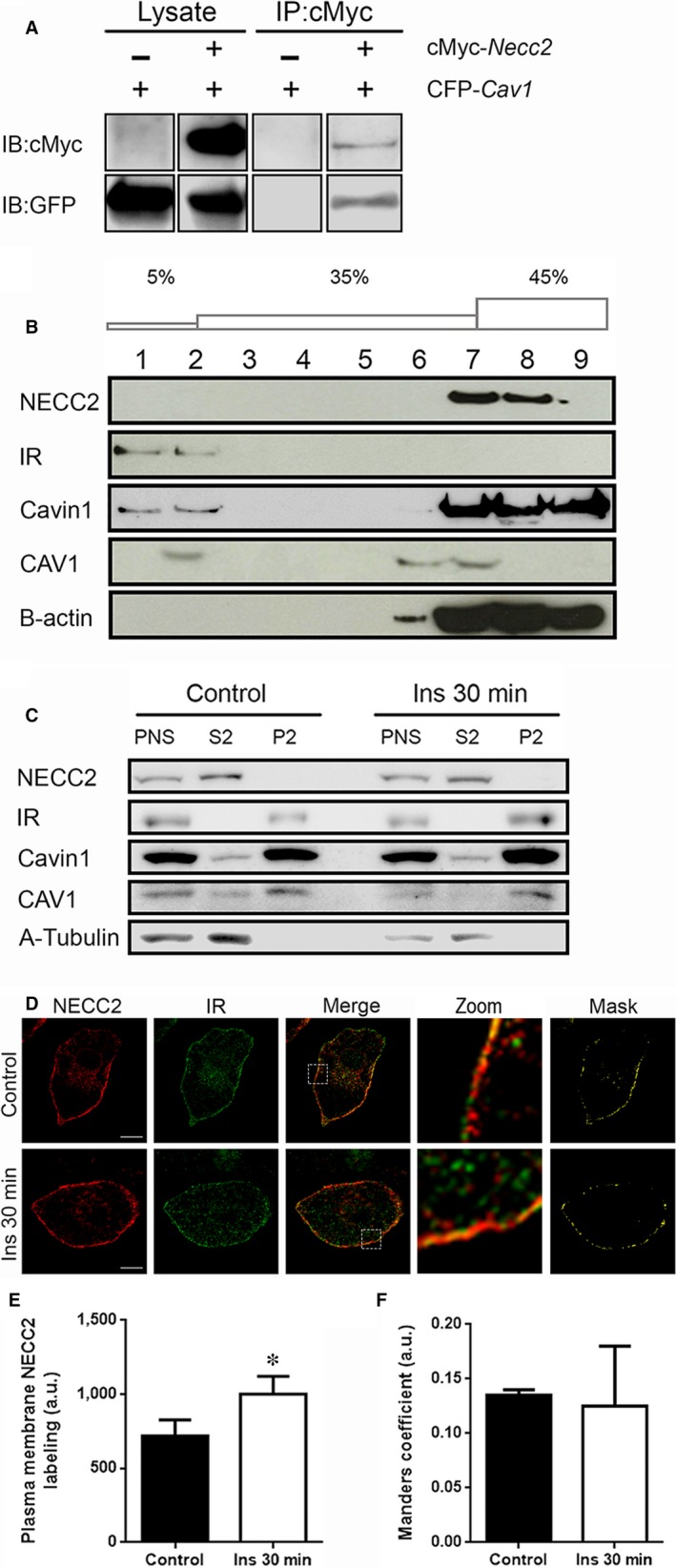
Association of NECC2 with caveolae and its regulation by insulin in 3T3‐L1 adipocytes. A, Direct interaction between NECC2 with CAV1 revealed by co‐immunoprecipitation assay. Both lysates and immunoprecipitates (IP) were subjected to immunoblotting with anti‐cMyc and anti‐GFP antibodies. For each antibody, lanes were run on the same gel but were not contiguous (Figure S2). B, Caveolae‐enriched membranes from 3T3‐L1 adipocytes were isolated by using a detergent‐free method based on a discontinuous sucrose gradient (5‐35‐45% w/v). Distribution of endogenous NECC2, insulin receptor (IR), Cavin1, CAV1 and B‐actin were assayed by immunoblot. C, Post‐nuclear supernatant (PNS), cytosolic (S2) and crude membrane (P2) fractions from 3T3‐L1 adipocytes under basal conditions and treated with insulin (100 nmol/L) for 30 min were obtained by subcellular fractionation as described in Methods. Distribution of endogenous NECC2, IR, Cavin1, CAV1 and A‐Tubulin were analysed by immunoblot. All the experiments were repeated at least twice to confirm the results. D, Confocal microscope images of 3T3‐L1 adipocytes under basal conditions (top panels) or treated with insulin (100 nmol/L) for 30 min (bottom panels). After treatment, cells were co‐immunostained for NECC2 (red) and insulin receptor (IR) (green). Magnified insets (zoom) and binary masks are shown at the two most right panels. Scale bar 10 μm. E, Quantification of NECC2 immunosignal at the membrane level in untreated cells (Control) and in cells exposed to 100 nmol/L insulin (Ins 30 min). NECC2 immunolabeling was quantified for each experimental condition in at least 8 cells (n = 2), and expressed as arbitrary units (a.u.). (F) Mander′s coefficient (between NECC2 and IR) was calculated to quantify the degree of colocalization and represented as the mean ± SEM of at least 12 cells per experimental group (n = 2), and expressed as arbitrary units (a.u.). The graphs represent the mean ± SEM. Data were analysed for significance using paired‐samples *t* test. *P <* *0.05 *vs* untreated cells (Control)

We further examine the association of NECC2 with CAV1 by co‐immunoprecipitation analysis, which revealed the interaction between these two proteins (Figure 3A and Figure S2). However, when we employed a detergent‐free method to isolate caveolin‐enriched membrane fractions,^18^, ^23^ we found that endogenous NECC2, at least the variant identified by the anti‐NECC2 antibody employed herein, did not co‐migrate with CAV1, Cavin1 or IR to “buoyant” fractions (Figure 3B). Notably, the Na_2_CO_3_ lysis buffer employed in this protocol for protein isolation maintains integral but not soluble and peripheral membrane proteins.^28^ In all, these results suggested that NECC2 may be peripherally bound to cell membranes. However, immunoblot analysis of post‐nuclear supernatant (PNS), cytosolic (S2) and crude membrane (P2) fractions from 3T3‐L1 adipocytes indicated that NECC2 is present in the soluble fraction (Figure 3C). These observations partially differ from those obtained in PC12 cells, wherein a low amount was also detected in association with membrane fractions, likely corresponding to an alternative NECC2 variant 18 that seems to be absent in 3T3‐L1 adipocytes. Indeed, the cytosolic distribution of NECC2 was observed in 3T3‐L1 adipocytes both under basal conditions and after short‐term exposure (30 minutes) to insulin (Figure 3C).

### 3.3 Insulin regulates NECC2 distribution in 3T3‐L1 adipocytes

We also investigated the intracellular distribution of NECC2 in 3T3‐L1 adipocytes after insulin stimulation. Interestingly, exposure to insulin (100 nmol/L, 30 minutes) increased NECC2 accumulation at the adipocyte surface (*P *=* *0.023) (Figure 3D and E).

Given the localization of the IR to adipocyte caveolae,^29^ we next explored the distribution of both NECC2 and IR at basal conditions and after a 30‐minute exposure to insulin (100 nmol/L). Examination by confocal microscopy revealed a high degree of overlap between NECC2 and IR immunosignals at the plasma membrane under basal conditions (Figure 3D). Short‐term stimulation with insulin did not alter the colocalization rate between NECC2 and IR (MC = 0.135 ± 0.005 *vs* 0.125 ± 0.055, *P *=* *0.876) (Figure 3F).

### 3.4 NECC2 regulates insulin signalling in adipocytes

**Figure 4:**
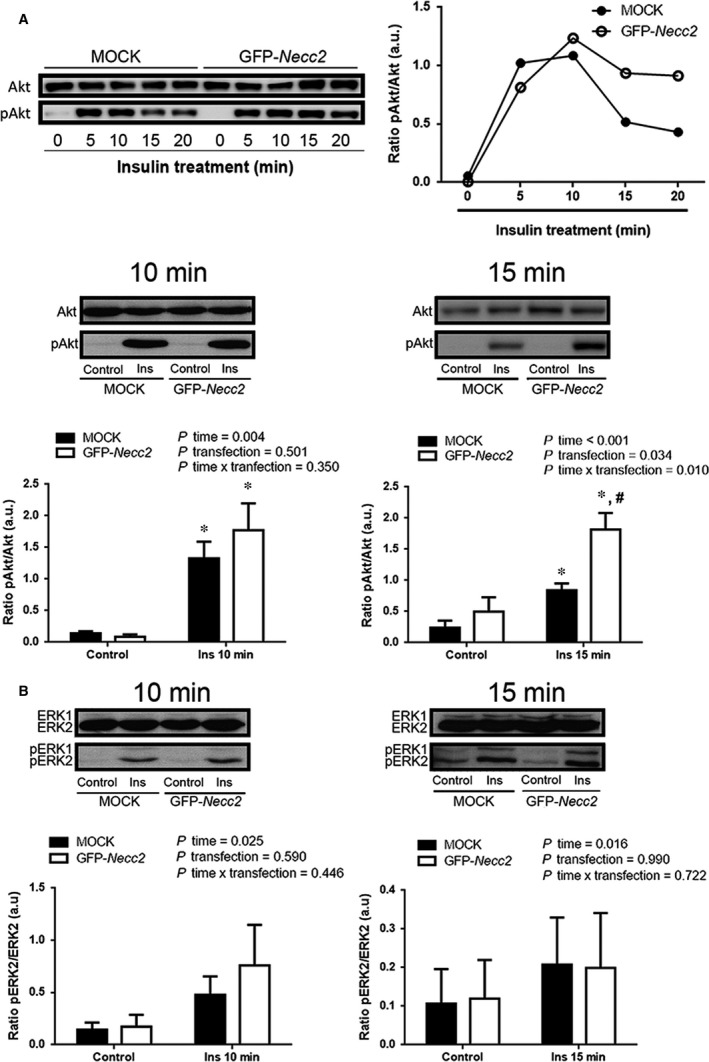
Effect of NECC2 overexpression on insulin signalling pathways. Differentiated 3T3‐L1 cells transiently transfected with GFP‐*Necc2*, or the empty vector (MOCK) were exposed for 2 h to serum‐low differentiation media before insulin stimulation (Ins, 100 nmol/L) for 10 or 15 min. Whole cell protein extracts were then subjected to immunoblot with Akt and phospho‐Akt (pAkt) antibodies (A) or with ERK and phospho‐ERK (pERK) antibodies (B). Quantitative data were represented as ratio of pAkt *vs* Akt or pERK2 *vs* ERK2, respectively. Data represent the means ± SEM of at least three independent experiments. Data were analysed using paired‐samples *t* test, independent samples *t* test and RM‐ANOVA was used to calculate the time effect (*P* time), transfection effect (*P* transfection) and the time x transfection interaction (*P* time × transfection). *P <* *0.05 *vs* corresponding untreated cells (control); ^#^ *P *<* *0.05 *vs* MOCK cells after insulin treatment

Based on our microscopic observations and given the role of caveolae as platforms for insulin signalling, we next investigated the effect of manipulating NECC2 expression levels on insulin intracellular mediators (ie ERK1/2 and Akt^3^) at different times after insulin exposure. These were selected on the basis of our previous studies on PC12 cells showing that NECC2 acts on ERK after long‐term exposure to NGF.^18^ To increase NECC2 expression, 3T3‐L1 adipocytes were electroporated with an expression vector coding for GFP‐*Necc2*, which enhanced NECC2 content by 59% (Figure S4a). In preliminary time‐course experiments, we observed that expression of GFP‐*Necc2* in 3T3‐L1 adipocytes resulted in a more sustained Akt phosphorylation rate as compared to mock‐transfected cells (Figure 4A). Indeed, analysis of Akt activation (pAkt/total Akt) upon insulin treatment revealed that the Akt phosphorylation rate was above baseline levels in both GFP‐*Necc2*‐ and mock‐transfected cells at 10 minutes (*P *<* *0.05). However, though Akt remained phosphorylated in both experimental groups after 15 minutes of insulin stimulation, pAkt/total Akt ratio in GFP‐*Necc2* expressing cells was twice that observed in insulin‐treated, mock‐transfected cells at 15 minutes (*P *=* *0.007) (Figure 4A). On the other hand, insulin‐induced ERK1/2 phosphorylation was not modified at any time point analysed in cells with increased expression of NECC2 compared to mock‐transfected cells (Figure 4B and Figure S4b for ERK2; Figure S5a to c for ERK1).

**Figure 5:**
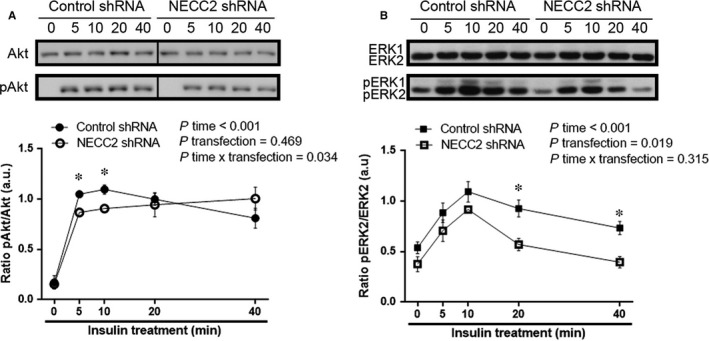
Effect of endogenous NECC2 silencing on insulin signalling pathways. (A and B) 3T3‐L1 adipocytes were transiently transfected with a shRNA for *Necc2* (NECC2 shRNA), or the empty vector (Control shRNA), pre‐treated 2 h with serum‐low differentiation media and treated with insulin (100 nmol/L) during the indicated time points. Immunoblotting were carried out with whole cells lysates and exposed to Akt and phospho‐Akt (pAkt) antibodies (A) or to ERK and phospho‐ERK (pERK) antibodies (B). Lanes were run on the same gel but were non‐contiguous in the Akt blot. Cropping line is used in the figure. Quantitative data were represented as ratio of pAkt *vs* Akt or pERK2 *vs* ERK2, respectively. Data represent the mean ± SEM of four independent experiments. Data were analysed using independent samples *t* test and RM‐ANOVA was used to calculate the time effect (*P* time), transfection effect (*P* transfection) and the time × transfection interaction (*P* time × transfection). *P <* *0.05 vs corresponding time point of control shRNA cells

We also examined the effect of NECC2 silencing on Akt and ERK1/2 using an expression vector coding a shRNA for *Necc2*, which decreased NECC2 protein content by 44% (Figure S1b‐c). Down‐regulation of NECC2 diminished both Akt and ERK2 phosphorylation (Figure 5). Specifically, Akt activity was significantly lower at 5 and 10 minutes of exposure to insulin in *Necc2‐*silenced cells than in control cells (*P *<* *0.05) (Figure 5A). ERK2 phosphorylation rate was also decreased in silenced cells at all the time points tested, although differences between NECC2 shRNA‐ and control shRNA‐treated cells reached statistical significance after 20 and 40 minutes of insulin stimulation (*P *<* *0.05) (Figure 5B). No differences were observed for ERK1 in these experiments (Figure S5d).

Neither NECC2 overexpression nor silencing modified CAV1 or IR content in 3T3‐L1 cells (Figure S6).

### 3.5 NECC2 expression in adipocytes is modulated in response to obesity and metabolic disturbances

**Figure 6:**
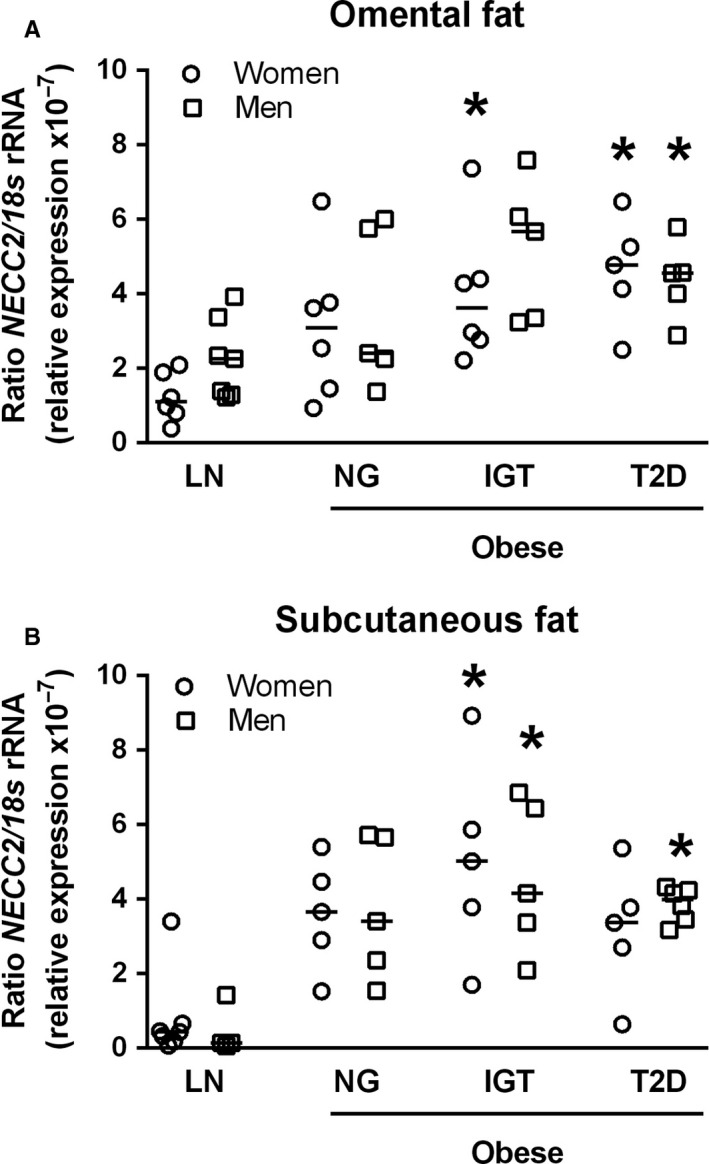
Assessment of NECC2 expression levels in relation to obesity and insulin resistance. (A and B). Omental and subcutaneous white adipose tissue samples were obtained from subjects with different degrees of obesity and/or insulin resistance [lean (LN); 6 women and 7 men in omental fat and 7 women and 4 men in subcutaneous fat], obese normoglycemic (NG; 6 women and 5 men in omental fat and 5 women and 5 men in subcutaneous fat), obese with impaired glucose tolerance (IGT; 6 women and 5 men in omental fat and 5 women and 5 men in subcutaneous fat) and obese type 2 diabetic (T2D; 5 women and 5 men in omental fat and 5 women and 6 men in subcutaneous fat) patients. *NECC2* gene expression was evaluated by RT‐PCR using specific primers for human NECC2. The expression of *18s* rRNA in each sample was evaluated as an internal housekeeping gene. Results are expressed in a dot plot format, which represents the individual data and the median. Data were analysed using One‐Way ANOVA. *P <* *0.05 *vs* sex‐related lean subject

We next assessed the expression of NECC2 in both omental and subcutaneous adipose tissue from lean and obese subjects. Obese subjects were stratified into NG, IGT or T2D groups. RT‐PCR analysis showed that *NECC2* gene is expressed in both fat depots, and that transcript levels of this protein were significantly higher in obese than in lean subjects irrespective of the fat depot and sex of the individual (data not shown). When the mRNA data from the three groups of obese individuals were analysed separately, we observed that both obese women and men with T2D exhibited significantly higher *NECC2* mRNA levels in omental adipose tissue than lean subjects (*P *<* *0.05) (Figure 6A). Furthermore, IGT obese women showed increased *NECC2* transcript content with respect to lean women (*P *=* *0.033) (Figure 6A). In subcutaneous fat, IGT obese women and men showed significantly greater NECC2 gene expression than lean subjects (*P *<* *0.05) (Figure 6B). Moreover, obese men, but not women, with T2D exhibited higher NECC2 mRNA levels than their lean counterparts (*P *=* *0.001) (Figure 6B).

**Figure 7:**
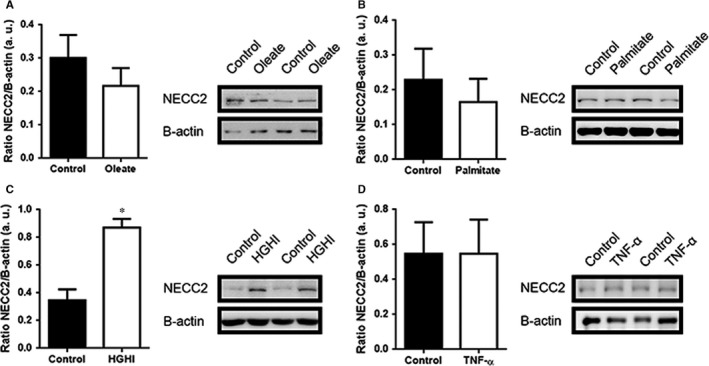
In vitro assessment of NECC2 expression levels in response to different metabolic insults. (A‐D) 3T3‐L1 adipocytes under different metabolic insults known to induce adipocyte hypertrophy and/or insulin resistance states [(A) 500 μmol/L oleate (n = 10) and (B) 500 μmol/L palmitate (n = 4) for 18 h, (C) high‐glucose (25 nmol/L)/ high‐insulin (100 nmol/L) (HGHI) for 24 h (n = 8) and (D) 5 nmol/L high‐TNF‐α for 24 h (n = 4)]. Data were expressed as arbitrary units (a.u.). Results represent the mean ± SEM. Data were analysed using paired‐samples *t* test. *P <* *0.05 *vs* Control

To further characterize NECC2 expression regulation in adipocytes, we performed *in vitro* studies including exposure of cells to fatty acids to induce adipocyte hypertrophy without (oleate; Figure 7A, Figure S6a‐c) or with accompanying insulin resistance (palmitate; Figure 7B), to hyperglycemia/hyperinsulinemia (high glucose/high insulin) (Figure 7C) or to inflammatory conditions (TNF‐α) (Figure 7D) as previously described by our group.^26^ These experiments revealed that long‐term exposure to high‐glucose/high‐insulin conditions increased by 2.5‐fold NECC2 protein levels in 3T3‐L1 adipocytes (Figure 7C).

## 4 DISCUSSION

Caveolae are distinctive membrane invaginations of particular relevance in adipocytes,^30^ where they comprise 50% of the cell surface.^31^ These specialized membrane domains are involved in a wide variety of cellular processes including vesicular transport, endocytosis and transcytosis 32 and act as cellular centres important in coordinating signalling complex formation.^33^ Caveolae are composed of structural and cytoskeletal proteins, scaffolding factors, cholesterol and sphingolipids and a number of signalling molecules [eg IR, MAPK cascade members, glucose transporter‐4 (GLUT‐4)], which are essential for caveolae formation, maintenance and functional organization.^9^, ^34^, ^35^, ^36^, ^37^, ^38^, ^39^

Our experiments in 3T3‐L1 cells revealed that NECC2 mRNA and protein content progressively increased during differentiation, reaching a maximal level in mature adipocytes. These data indicate that, as previously suggested for other long coiled‐coil proteins induced during adipogenesis, such as golgin‐160 and p115,^40^ NECC2 may play a role in adipocyte‐specific function.

We have previously shown that NECC2 associated to caveolae in neuroendocrine PC12 cells.^18^ Given this observation and the distribution of NECC2 immunosignal to the cell surface of 3T3‐L1 cells, we investigated the potential interaction between NECC2 and CAV1 by both confocal microscopy and immunoprecipitation studies in 3T3‐L1 adipocytes. These studies revealed that these proteins did interact and exhibited similar distributions patterns, supporting the colocalization of NECC2 and CAV1 at the adipocyte surface. NECC2 immunolabeling also showed a high degree of colocalization with the caveolae component, Cavin1,^30^ as well as with cortical actin, which has been shown to localize along the inner circumference of ring‐like caveolae clusters (ie caveolae rosettes), to regulate the dynamics and localization of CAV1 in these cholesterol‐enriched lipid rafts^41^, ^42^ and to promote insulin‐dependent GLUT‐4 translocation via the small GTP binding protein, TC10, a member of the Rho family.^43^ Accordingly, latrunculin B‐induced actin depolymerization decreased significantly the overlap between NECC2 signal and phalloidin (ie cortical actin) in 3T3‐L1 adipocytes. However, no changes were observed in the degree of overlap between NECC2 and CAV1 in latrunculin B‐treated adipocytes, suggesting that NECC2 association to caveolae is not dependent upon F‐actin. These results are in line with previous studies showing that exposure of 3T3‐L1 adipocytes to latrunculin B does not affect the integrity and abundance of caveolae.^27^, ^41^ Taken together, these data demonstrate that NECC2 is located to caveolae in 3T3‐L1 adipocytes, likely through its association with CAV1, as supported also by our silencing studies showing that CAV1 depletion impaired NECC2 accumulation at the cell surface. The interaction between NECC2 and CAV1 might be transient, since membrane cholesterol depletion by methyl‐β‐cyclodextrin, which disrupts the lipid organization of caveolar domains,^44^ reduced the colocalization index between NECC2 and CAV1. Nevertheless, studies by total internal reflection fluorescence (TIRF) microscopy are needed to assess more accurately the precise localization and dynamics of NECC2 near the plasma membrane with respect to other components of the adipocyte caveolae.

In this line, detergent‐free extraction of caveolae‐enriched membranes and subcellular fractionation revealed that NECC2 behaves as a soluble protein that is present at the cytosolic face of caveolae, which is consistent with our previous observations in PC12 cells.^18^ In this regard, cavins are soluble proteins with predicted coiled‐coil structures and basic residues that are abundantly present at the cytosolic face of caveolae.^11^, ^45^ Notably, it has been observed that long coiled‐coil proteins at the Golgi apparatus, the *golgins*, undergo dissociation/association cycles between the cytosol and Golgi membranes to which they anchor through binding to Golgi‐associated proteins.^46^ Besides interacting with CAV1, NECC2 might reside in caveolae via interaction with other caveolae‐associated proteins. In particular, STRING database search for NECC2 interactors predicted the association of this protein with other proteins potentially linked, directly or indirectly, to caveolae (YIPF5, CDC37). In this regard, N‐myristoylation sites present in proteins such as NECC2 have been proposed to allow for weak protein‐protein and protein‐lipid interactions 47 and, thus, play an essential role in membrane targeting and protein‐protein interactions.^48^

Caveolae play a pivotal role in the spatial compartmentalization of insulin signalling.^49^ The activation of this signalling network is triggered by binding of insulin to its receptor, which is localized to caveolae,^27^, ^29^ via interaction with the underlying cytoskeletal proteins enriched in these microdomains.^27^ Herein, we observed a significant degree of overlap between NECC2 and IR immunosignals at the cell surface. Several studies have shown that this receptor may undergo internalization after insulin stimulation.^50^, ^51^ However, we observed that the colocalization rate between NECC2 and IR did not decrease in 3T3‐L1 adipocytes exposed to insulin, but an increase in NECC2 accumulation at the cell surface, suggesting that this hormone induces the recruitment of NECC2 to the cell surface. Similar findings have been reported for human insulin receptor substrate‐1 (IRS1), a critical adaptor protein in the insulin signalling pathway, which also accumulates at the cell surface after an acute insulin challenge.^37^, ^52^ As mentioned earlier, we have previously shown that NECC2 colocalized with the NGF receptor, TrkA, at caveolae in PC12 cells and that the amount of NECC2 at the cell surface increased upon exposure to this growth factor.^18^ Taken together, our data in both adipocytes and neuroendocrine cells suggest that NECC2 at caveolae may act as a common mediator of growth factor signalling (insulin and NGF, respectively), likely through its physical and/or functional interaction with growth factor receptors (IR and TrkA, respectively).

Given that a cholesterol‐enriched lipid microenviroment is critical to allow IR to stimulate the PI3K/Akt pathway,^53^ and IR interaction with the cytoskeleton is required for proper ERK1/2 activation,^54^ we analysed the effect of modulating NECC2 content on both IR‐activated downstream effectors in adipocytes. Thus, overexpression of NECC2 in 3T3‐L1 cells produced a sustained stimulation of insulin‐activated phosphorylation of Akt. Notably, NECC2 overexpression had no effect on IR‐dependent activation of ERK1/2, which is also mediated via insulin binding to IR,^55^ but has been shown to occur during IR receptor internalization in endosomes.^51^ On the other hand, down‐regulation of NECC2 impaired insulin‐induced phosphorylation of both Akt and the major ERK isoform, ERK2.^56^ These results, together with NECC2 overexpression data, suggest that appropriate NECC2 expression levels are required for proper IR activity, both at the plasma membrane and the endosomes. Our previous studies in PC12 cells showed that both NECC2 overexpression and silencing altered NGF‐activated ERK phosphorylation, whereas Akt phosphorylation was unaffected.^18^ Together, these observations strongly support the contribution of NECC2 to maintain the function of caveolae as signalling platforms by selectively regulating, in a cell‐type dependent manner, specific signalling cascades.

Based on our findings on NECC2 in 3T3‐L1 adipocytes and given the close relationship between human obesity and insulin resistance/T2D,^57^ we next examined NECC2 in human adipose tissue in subjects with different glucose metabolism states. We found that, irrespective of the fat depot and sex of the individuals, obesity was associated with an increase in *NECC2* expression, which might represent an adaptive response to overcome the alterations occurring in conditions of increased fat mass. Nevertheless, although we observed a tendency towards higher *NECC2* mRNA expression in obese normoglycemic men and women compared to lean subjects in both fat depots, only when obese individuals showed insulin resistance (IGT for both depots and genders; T2D for both depots only in men) these levels reached statistical significance as compared to lean subjects. Notably, these changes parallel those previously reported for CAV1 in human adipose tissue.^58^ Interestingly, there is evidence supporting that sustained activation of Akt, as occurs in 3T3‐L1 adipocytes with enhanced NECC2 expression levels, leads to a negative feedback of insulin signalling and, therefore, insulin resistance.^25^ Together, these and our morphological and functional data in 3T3‐L1 cells may suggest a role for NECC2 in obesity and the development of glucose metabolism derangements.

Finally, in order to get insights into the mechanisms regulating NECC2 expression in adipose tissue, we assessed the expression of this protein in *in vitro* models of adipocyte hypertrophy and/or insulin resistance.^26^, ^59^, ^60^ We observed that induction of insulin resistance by exposure to HGHI significantly increased NECC2 protein levels, which is in accordance with the changes observed in human adipose tissue. Unexpectedly, however, neither palmitate nor TNF‐α, which we have previously shown to evoke insulin resistance,^26^ or oleate‐induced hypertrophy significantly modified NECC2 content in 3T3‐L1 cells. These results are in line with previous observations from our laboratory and other groups indicating that different insults operating in obesity (ie cytokines, lipids, glucose) may activate distinct subcellular responses in adipocytes.^26^, ^59^, ^60^ In this scenario, it is tempting to speculate that the up‐regulation of NECC2 observed in insulin‐resistant obese individuals results from the activation of obesity‐triggered cellular stress responses.

In conclusion, our data provide the first demonstration of the association of a long coiled‐coil protein, NECC2, to adipocyte caveolae and identify this protein as a potential regulator of the insulin/IR system in adipocytes. In addition, they suggest that NECC2 up‐regulation in human adipose tissue may contribute, at least in part, to the development of insulin resistance in obesity.

## CONFLICT OF INTEREST

The authors confirm that there are no conflicts of interest.

## References

[1] Smith U . Impaired (‘diabetic’) insulin signaling and action occur in fat cells long before glucose intolerance–is insulin resistance initiated in the adipose tissue? Int J Obes Relat Metab Disord. 2002;26:897‐904. 12080441 10.1038/sj.ijo.0802028

[2] DeFronzo RA , Tripathy D . Skeletal muscle insulin resistance is the primary defect in type 2 diabetes. Diabetes Care. 2009;32(Suppl 2):S157‐S163. 19875544 10.2337/dc09-S302 PMC2811436

[3] Taniguchi CM , Emanuelli B , Kahn CR . Critical nodes in signalling pathways: insights into insulin action. Nat Rev Mol Cell Biol. 2006;7:85‐96. 16493415 10.1038/nrm1837

[4] Siddle K . Signalling by insulin and IGF receptors: supporting acts and new players. J Mol Endocrinol. 2011;47:R1‐R10. 21498522 10.1530/JME-11-0022

[5] Saltiel AR , Pessin JE . Insulin signaling pathways in time and space. Trends Cell Biol. 2002;12:65‐71. 11849969 10.1016/s0962-8924(01)02207-3

[6] Lamaze C , Tardif N , Dewulf M , Vassilopoulos S , Blouin CM . The caveolae dress code: structure and signaling. Curr Opin Cell Biol. 2017;47:117‐125. 28641181 10.1016/j.ceb.2017.02.014

[7] Cohen AW , Hnasko R , Schubert W , Lisanti MP . Role of caveolae and caveolins in health and disease. Physiol Rev. 2004;84:1341‐1379. 15383654 10.1152/physrev.00046.2003

[8] Fruhbeck G . The Sir David Cuthbertson Medal Lecture. Hunting for new pieces to the complex puzzle of obesity. Proc Nutr Soc. 2006;65:329‐347. 17181900 10.1017/s0029665106005106

[9] Parton RG , del Pozo MA . Caveolae as plasma membrane sensors, protectors and organizers. Nat Rev Mol Cell Biol. 2013;14:98‐112. 23340574 10.1038/nrm3512

[10] Fruhbeck G , Lopez M , Dieguez C . Role of caveolins in body weight and insulin resistance regulation. Trends Endocrinol Metab. 2007;18:177‐182. 17433707 10.1016/j.tem.2007.04.001

[11] Kovtun O , Tillu VA , Ariotti N , Parton RG , Collins BM . Cavin family proteins and the assembly of caveolae. J Cell Sci. 2015;128:1269‐1278. 25829513 10.1242/jcs.167866 PMC4379724

[12] Scherer PE , Lisanti MP , Baldini G , Sargiacomo M , Mastick CC , Lodish HF . Induction of caveolin during adipogenesis and association of GLUT4 with caveolin‐rich vesicles. J Cell Biol. 1994;127:1233‐1243. 7962086 10.1083/jcb.127.5.1233 PMC2120260

[13] Nystrom FH , Chen H , Cong LN , Li Y , Quon MJ . Caveolin‐1 interacts with the insulin receptor and can differentially modulate insulin signaling in transfected Cos‐7 cells and rat adipose cells. Mol Endocrinol. 1999;13:2013‐2024. 10598578 10.1210/mend.13.12.0392

[14] Chidlow JH Jr , Sessa WC . Caveolae, caveolins, and cavins: complex control of cellular signalling and inflammation. Cardiovasc Res. 2010;86:219‐225. 20202978 10.1093/cvr/cvq075 PMC2856194

[15] Rose A , Meier I . Scaffolds, levers, rods and springs: diverse cellular functions of long coiled‐coil proteins. Cell Mol Life Sci. 2004;61:1996‐2009. 15316650 10.1007/s00018-004-4039-6 PMC11138566

[16] Steindler C , Li Z , Algarte M , et al. Jamip1 (marlin‐1) defines a family of proteins interacting with janus kinases and microtubules. J Biol Chem. 2004;279:43168‐43177. 15277531 10.1074/jbc.M401915200

[17] Cruz‐Garcia D , Vazquez‐Martinez R , Peinado JR , et al. Identification and characterization of two novel (neuro)endocrine long coiled‐coil proteins. FEBS Lett. 2007;581:3149‐3156. 17572408 10.1016/j.febslet.2007.06.002

[18] Diaz‐Ruiz A , Rabanal‐Ruiz Y , Travez A , et al. The long coiled‐coil protein NECC2 is associated to caveolae and modulates NGF/TrkA signaling in PC12 cells. PLoS One. 2013;8:e73668. 24040018 10.1371/journal.pone.0073668 PMC3765260

[19] Pulido MR , Diaz‐Ruiz A , Jimenez‐Gomez Y , et al. Rab18 dynamics in adipocytes in relation to lipogenesis, lipolysis and obesity. PLoS One. 2011;6:e22931. 21829560 10.1371/journal.pone.0022931 PMC3145781

[20] American Diabetes Association . Classification and diagnosis of diabetes. Diabetes Care. 2015;38(Suppl 1):S8‐S16. 10.2337/dc15-S005 25537714

[21] Catalan V , Gomez‐Ambrosi J , Rotellar F , et al. Validation of endogenous control genes in human adipose tissue: relevance to obesity and obesity‐associated type 2 diabetes mellitus. Horm Metab Res. 2007;39:495‐500. 17611901 10.1055/s-2007-982502

[22] Jimenez‐Gomez Y , Cruz‐Teno C , Rangel‐Zuniga OA , et al. Effect of dietary fat modification on subcutaneous white adipose tissue insulin sensitivity in patients with metabolic syndrome. Mol Nutr Food Res. 2014;58:2177‐2188. 25044988 10.1002/mnfr.201300901

[23] Moreno‐Castellanos N , Rodriguez A , Rabanal‐Ruiz Y , et al. The cytoskeletal protein septin 11 is associated with human obesity and is involved in adipocyte lipid storage and metabolism. Diabetologia. 2017;60:324‐335. 27866222 10.1007/s00125-016-4155-5

[24] Rodriguez‐Pacheco F , Vazquez‐Martinez R , Martinez‐Fuentes AJ , et al. Resistin regulates pituitary somatotrope cell function through the activation of multiple signaling pathways. Endocrinology. 2009;150:4643‐4652. 19589870 10.1210/en.2009-0116

[25] Jimenez‐Gomez Y , Mattison JA , Pearson KJ , et al. Resveratrol improves adipose insulin signaling and reduces the inflammatory response in adipose tissue of rhesus monkeys on high‐fat, high‐sugar diet. Cell Metab. 2013;18:533‐545. 24093677 10.1016/j.cmet.2013.09.004 PMC3832130

[26] Diaz‐Ruiz A , Guzman‐Ruiz R , Moreno NR , et al. Proteasome dysfunction associated to oxidative stress and proteotoxicity in adipocytes compromises insulin sensitivity in human obesity. Antioxid Redox Signal. 2015;23:597‐612. 25714483 10.1089/ars.2014.5939 PMC4554552

[27] Foti M , Porcheron G , Fournier M , Maeder C , Carpentier JL . The neck of caveolae is a distinct plasma membrane subdomain that concentrates insulin receptors in 3T3‐L1 adipocytes. Proc Natl Acad Sci U S A. 2007;104:1242‐1247. 17227843 10.1073/pnas.0610523104 PMC1783101

[28] Peiro S , Comella JX , Enrich C , Martin‐Zanca D , Rocamora N . PC12 cells have caveolae that contain TrkA. Caveolae‐disrupting drugs inhibit nerve growth factor‐induced, but not epidermal growth factor‐induced, MAPK phosphorylation. J Biol Chem. 2000;275:37846‐37852. 10982788 10.1074/jbc.M000487200

[29] Gustavsson J , Parpal S , Karlsson M , et al. Localization of the insulin receptor in caveolae of adipocyte plasma membrane. FASEB J. 1999;13:1961‐1971. 10544179

[30] Pilch PF , Liu L . Fat caves: caveolae, lipid trafficking and lipid metabolism in adipocytes. Trends Endocrinol Metab. 2011;22:318‐324. 21592817 10.1016/j.tem.2011.04.001 PMC3149783

[31] Thorn H , Stenkula KG , Karlsson M , et al. Cell surface orifices of caveolae and localization of caveolin to the necks of caveolae in adipocytes. Mol Biol Cell. 2003;14:3967‐3976. 14517311 10.1091/mbc.E03-01-0050 PMC206992

[32] Schnitzer JE , Allard J , Oh P . NEM inhibits transcytosis, endocytosis, and capillary permeability: implication of caveolae fusion in endothelia. Am J Physiol. 1995;268:H48‐H55. 7840297 10.1152/ajpheart.1995.268.1.H48

[33] Smart EJ , Graf GA , McNiven MA , et al. Caveolins, liquid‐ordered domains, and signal transduction. Mol Cell Biol. 1999;19:7289‐7304. 10523618 10.1128/mcb.19.11.7289 PMC84723

[34] Hayer A , Stoeber M , Bissig C , Helenius A . Biogenesis of caveolae: stepwise assembly of large caveolin and cavin complexes. Traffic. 2010;11:361‐382. 20070607 10.1111/j.1600-0854.2009.01023.x

[35] Richter T , Floetenmeyer M , Ferguson C , et al. High‐resolution 3D quantitative analysis of caveolar ultrastructure and caveola‐cytoskeleton interactions. Traffic. 2008;9:893‐909. 18397183 10.1111/j.1600-0854.2008.00733.x

[36] Okamoto T , Schlegel A , Scherer PE , Lisanti MP . Caveolins, a family of scaffolding proteins for organizing “preassembled signaling complexes” at the plasma membrane. J Biol Chem. 1998;273:5419‐5422. 9488658 10.1074/jbc.273.10.5419

[37] Karlsson M , Thorn H , Danielsson A , et al. Colocalization of insulin receptor and insulin receptor substrate‐1 to caveolae in primary human adipocytes. Cholesterol depletion blocks insulin signalling for metabolic and mitogenic control. Eur J Biochem. 2004;271:2471‐2479. 15182363 10.1111/j.1432-1033.2004.04177.x

[38] Engelman JA , Chu C , Lin A , et al. Caveolin‐mediated regulation of signaling along the p42/44 MAP kinase cascade in vivo. A role for the caveolin‐scaffolding domain. FEBS Lett. 1998;428:205‐211. 9654135 10.1016/s0014-5793(98)00470-0

[39] Yuan T , Hong S , Yao Y , Liao K . Glut‐4 is translocated to both caveolae and non‐caveolar lipid rafts, but is partially internalized through caveolae in insulin‐stimulated adipocytes. Cell Res. 2007;17:772‐782. 17846641 10.1038/cr.2007.73

[40] Williams D , Hicks SW , Machamer CE , Pessin JE . Golgin‐160 is required for the Golgi membrane sorting of the insulin‐responsive glucose transporter GLUT4 in adipocytes. Mol Biol Cell. 2006;17:5346‐5355. 17050738 10.1091/mbc.E06-05-0386 PMC1679696

[41] Kanzaki M , Pessin JE . Caveolin‐associated filamentous actin (Cav‐actin) defines a novel F‐actin structure in adipocytes. J Biol Chem. 2002;277:25867‐25869. 12039946 10.1074/jbc.C200292200

[42] Mundy DI , Machleidt T , Ying YS , Anderson RG , Bloom GS . Dual control of caveolar membrane traffic by microtubules and the actin cytoskeleton. J Cell Sci. 2002;115:4327‐4339. 12376564 10.1242/jcs.00117

[43] Bridges D , Chang L , Lodhi IJ , Clark NA , Saltiel AR . TC10 is regulated by caveolin in 3T3‐L1 adipocytes. PLoS One. 2012;7:e42451. 22900022 10.1371/journal.pone.0042451 PMC3416860

[44] Shigematsu S , Watson RT , Khan AH , Pessin JE . The adipocyte plasma membrane caveolin functional/structural organization is necessary for the efficient endocytosis of GLUT4. J Biol Chem. 2003;278:10683‐10690. 12496259 10.1074/jbc.M208563200

[45] Shvets E , Bitsikas V , Howard G , Hansen CG , Nichols BJ . Dynamic caveolae exclude bulk membrane proteins and are required for sorting of excess glycosphingolipids. Nat Commun. 2015;6:6867. 25897946 10.1038/ncomms7867 PMC4410672

[46] Gillingham AK , Munro S . Finding the Golgi: Golgin coiled‐coil proteins show the way. Trends Cell Biol. 2016;26:399‐408. 26972448 10.1016/j.tcb.2016.02.005

[47] Farazi TA , Waksman G , Gordon JI . The biology and enzymology of protein N‐myristoylation. J Biol Chem. 2001;276:39501‐39504. 11527981 10.1074/jbc.R100042200

[48] Rowe DC , McGettrick AF , Latz E , et al. The myristoylation of TRIF‐related adaptor molecule is essential for Toll‐like receptor 4 signal transduction. Proc Natl Acad Sci U S A. 2006;103:6299‐6304. 16603631 10.1073/pnas.0510041103 PMC1458872

[49] Kabayama K , Sato T , Saito K , et al. Dissociation of the insulin receptor and caveolin‐1 complex by ganglioside GM3 in the state of insulin resistance. Proc Natl Acad Sci U S A. 2007;104:13678‐13683. 17699617 10.1073/pnas.0703650104 PMC1949342

[50] Robert Carpentier JL . Robert Feulgen Prize Lecture 1993. The journey of the insulin receptor into the cell: from cellular biology to pathophysiology. Histochemistry. 1993;1993(100):169‐184. 10.1007/BF00269090 8244769

[51] Boothe T , Lim GE , Cen H , et al. Inter‐domain tagging implicates caveolin‐1 in insulin receptor trafficking and Erk signaling bias in pancreatic beta‐cells. Mol Metabol. 2016;5:366‐378. 10.1016/j.molmet.2016.01.009 PMC4837300 27110488

[52] Stenkula KG , Thorn H , Franck N , et al. Human, but not rat, IRS1 targets to the plasma membrane in both human and rat adipocytes. Biochem Biophys Res Commun. 2007;363:840‐845. 17905199 10.1016/j.bbrc.2007.09.065

[53] Parpal S , Karlsson M , Thorn H , Stralfors P . Cholesterol depletion disrupts caveolae and insulin receptor signaling for metabolic control via insulin receptor substrate‐1, but not for mitogen‐activated protein kinase control. J Biol Chem. 2001;276:9670‐9678. 11121405 10.1074/jbc.M007454200

[54] He HJ , Kole S , Kwon YK , Crow MT , Bernier M . Interaction of filamin A with the insulin receptor alters insulin‐dependent activation of the mitogen‐activated protein kinase pathway. J Biol Chem. 2003; 278:27096‐27104. 12734206 10.1074/jbc.M301003200

[55] Frojdo S , Vidal H , Pirola L . Alterations of insulin signaling in type 2 diabetes: a review of the current evidence from humans. Biochim Biophys Acta. 2009;1792:83‐92. 19041393 10.1016/j.bbadis.2008.10.019

[56] Busca R , Pouyssegur J , Lenormand P . ERK1 and ERK2 map kinases: specific roles or functional redundancy? Front Cell Develop Biol. 2016;4:53. 10.3389/fcell.2016.00053 PMC4897767 27376062

[57] Kahn BB , Flier JS . Obesity and insulin resistance. J Clin Invest. 2000;106:473‐481. 10953022 10.1172/JCI10842 PMC380258

[58] Catalan V , Gomez‐Ambrosi J , Rodriguez A , et al. Expression of caveolin‐1 in human adipose tissue is upregulated in obesity and obesity‐associated type 2 diabetes mellitus and related to inflammation. Clin Endocrinol (Oxf). 2008;68:213‐219. 17803693 10.1111/j.1365-2265.2007.03021.x

[59] Buren J , Liu HX , Lauritz J , Eriksson JW . High glucose and insulin in combination cause insulin receptor substrate‐1 and ‐2 depletion and protein kinase B desensitisation in primary cultured rat adipocytes: possible implications for insulin resistance in type 2 diabetes. Eur J Endocrinol. 2003;148:157‐167. 12534369 10.1530/eje.0.1480157

[60] Yeop Han C , Kargi AY , Omer M , et al. Differential effect of saturated and unsaturated free fatty acids on the generation of monocyte adhesion and chemotactic factors by adipocytes: dissociation of adipocyte hypertrophy from inflammation. Diabetes. 2010;59:386‐396. 19934003 10.2337/db09-0925 PMC2809975

