# Supplementary material for: The caveolae‐associated coiled‐coil protein, NECC2, regulates insulin signalling in Adipocytes
Source: J Cell Mol Med. 2018 Aug 30;22(11):5648–61. doi: 10.1111/jcmm.13840 (PMC6201366; doi:10.1111/jcmm.13840)
Supplement: Supplementary file 1 [file JCMM-22-5648-s001.doc]

**Figure S1.** Differentiation markers in 3T3-L1 cells and analysis of the specificity of anti-NECC2 antibody.

**
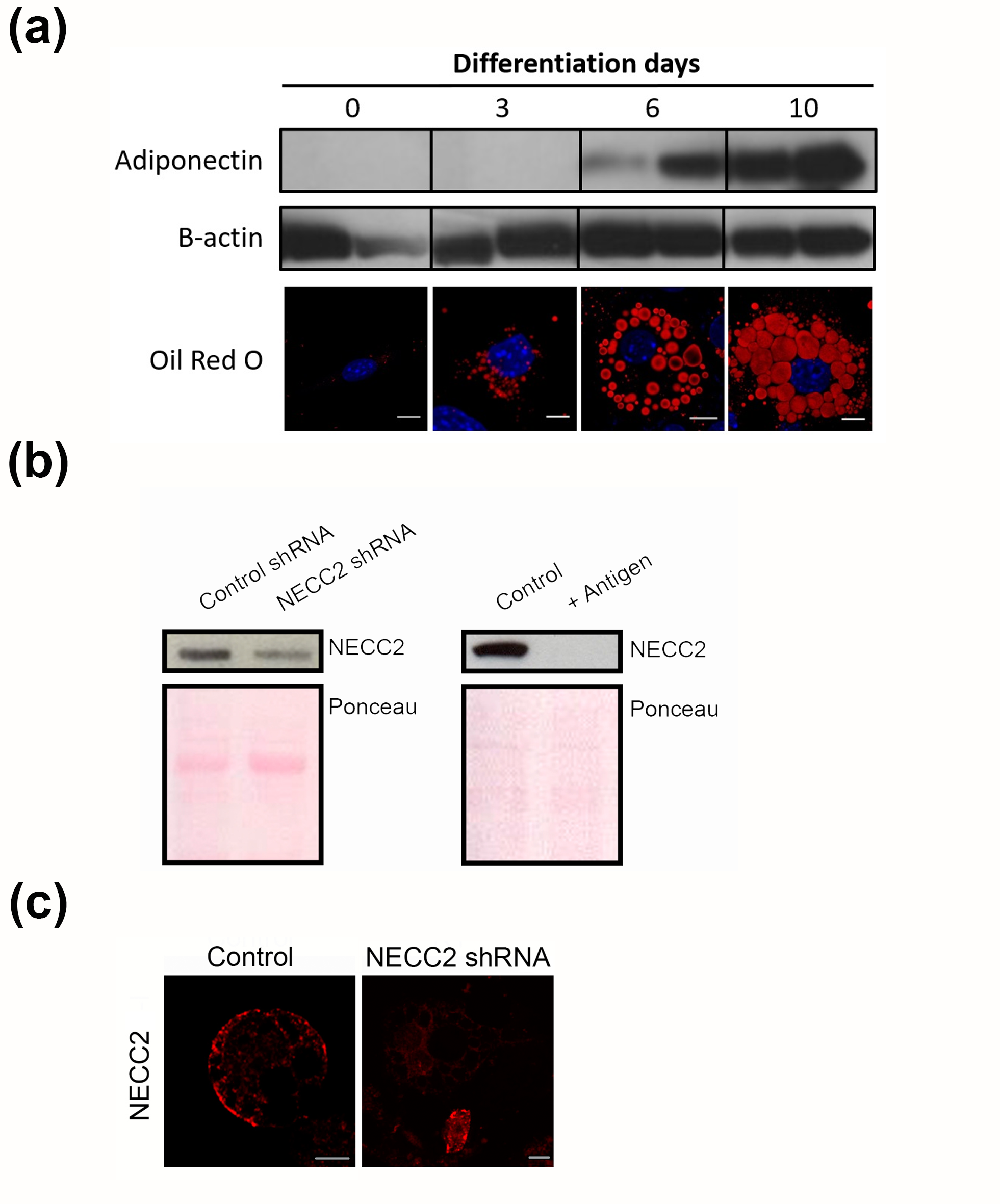
**

Immunoblot analysis of adiponectin protein content using anti-Adiponectin antibody during differentiation of 3T3-L1 cells into adipocytes. B-actin staining was used as load control. Lanes were run on the same gel (n=4). Bottom panels shows representative confocal images of 3T3-L1 adipocytes treated with Oil Red O (n =2). DAPI was used for nuclei labeling. Scale bar 10 μm (a). Immunoblot analysis of NECC2 protein content using anti-NECC2 antibody. Immunodetection was abolished in *Necc2* shRNA transfected cells (NECC2 shRNA) and after preabsorption of the antibody with the purified antigen. Ponceau staining was used for quantity protein controls. Lanes were run on the same gel (b). Immunocytochemistry analysis of NECC2 protein using anti-NECC2 antibody. Immunodetection was abolished in *Necc2* shRNA transfected cells (NECC2 shRNA) and after preabsorption of the antibody with the purified antigen (c). Scale bar 10 μm.
