# Supplementary material for: The caveolae‐associated coiled‐coil protein, NECC2, regulates insulin signalling in Adipocytes
Source: J Cell Mol Med. 2018 Aug 30;22(11):5648–61. doi: 10.1111/jcmm.13840 (PMC6201366; doi:10.1111/jcmm.13840)
Supplement: Supplementary file 2 [file JCMM-22-5648-s002.doc]

**Figure S2.** Western blot images from immunoblot analysis of immunoprecipitation experiments.

**
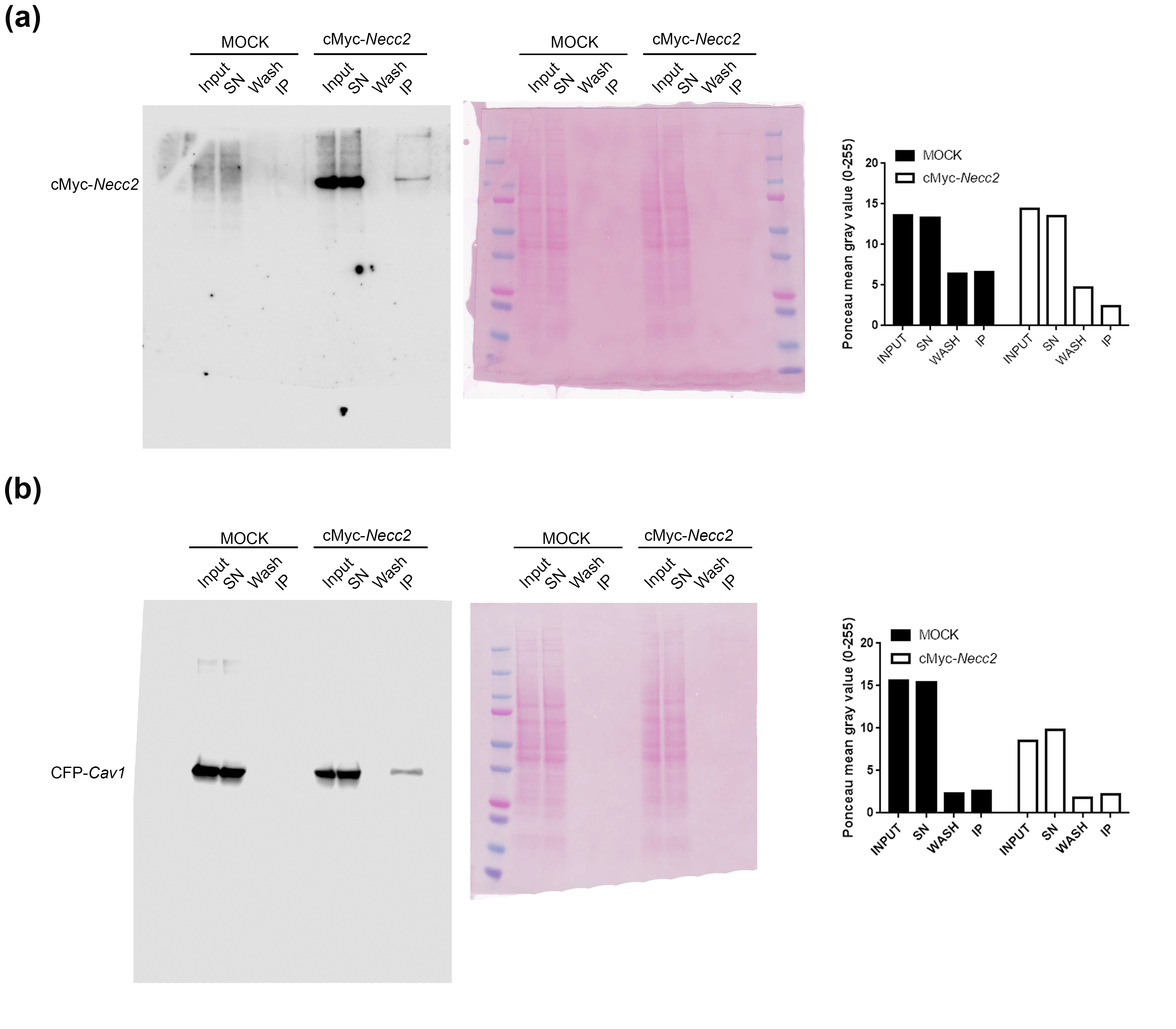
**

Uncropped Western blots and ponceau images related to Fig. 2d, corresponding to extracts from detection of c-Myc-NECC2 (a) and CFP-CAV1 (b) in double-transfected HEK-293 AD cells. The graphs show the quantification of Ponceau, and the results are expressed as mean ± SEM. SN: supernatant; IP: immunoprecipitates.
