# Supplementary material for: The caveolae‐associated coiled‐coil protein, NECC2, regulates insulin signalling in Adipocytes
Source: J Cell Mol Med. 2018 Aug 30;22(11):5648–61. doi: 10.1111/jcmm.13840 (PMC6201366; doi:10.1111/jcmm.13840)
Supplement: Supplementary file 3 [file JCMM-22-5648-s003.doc]

**Figure S3.** Analysis of NECC2 localization with protein excluded from caveolae, and validation of *Cav1* silencing.


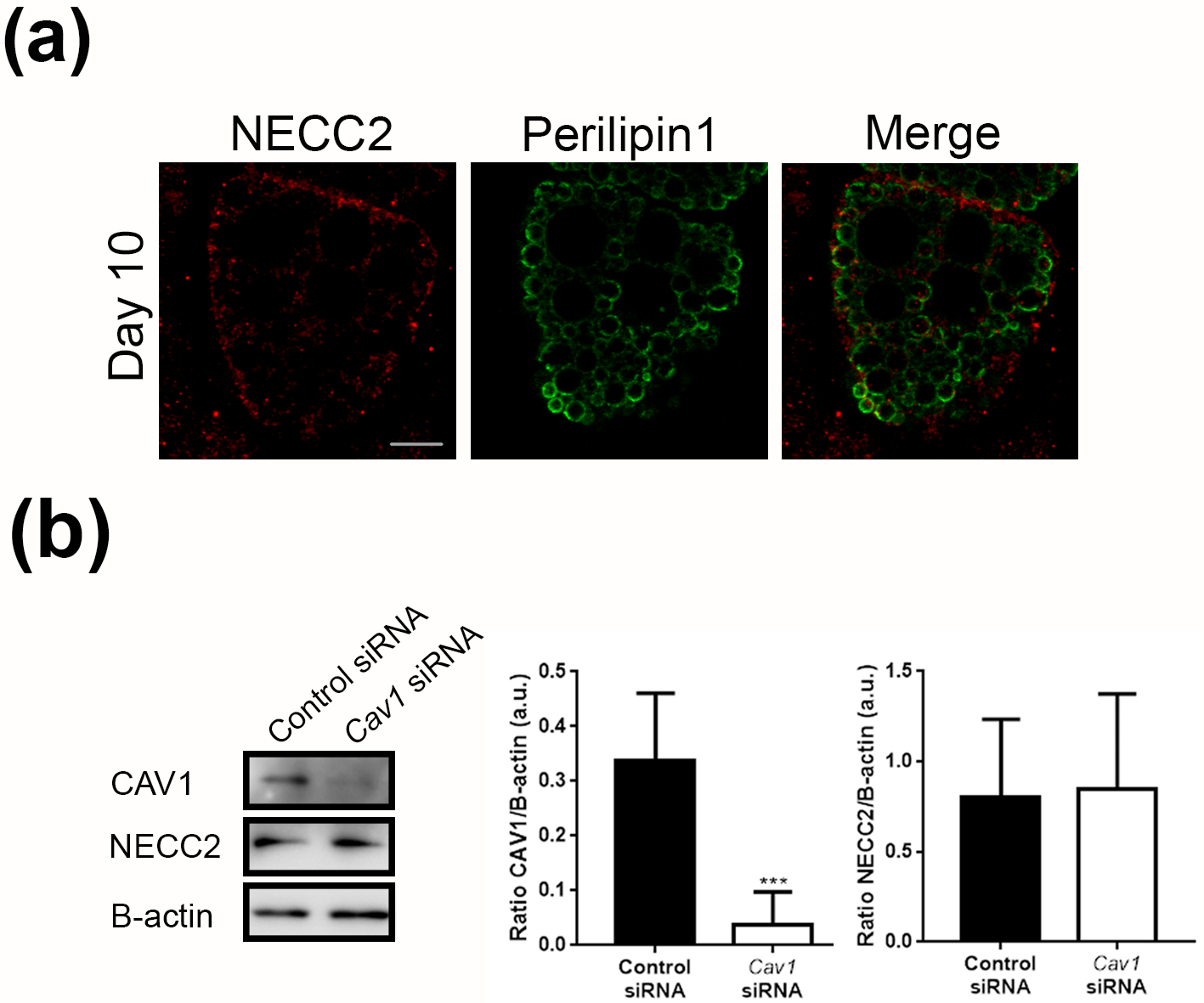


Comparison of the NECC2 immunosignal with the protein excluded from caveolae, Perilipin1, related to Fig. 2a. Representative confocal images are shown (n = 2). Scale bar 10 μm. (a). *Cav1* silencing, related to Fig. 2d. Whole cell protein extracts were subjected to immunoblot using caveolin 1 (CAV1), NECC2, and B-actin antibodies. The data are expressed as a ratio between target proteins and B-actin, and represent the means ± SEM of 5 independent experiments. Data were analyzed using independent samples t test and expressed as arbitrary units (a.u.) (b).
