# Supplementary material for: The caveolae‐associated coiled‐coil protein, NECC2, regulates insulin signalling in Adipocytes
Source: J Cell Mol Med. 2018 Aug 30;22(11):5648–61. doi: 10.1111/jcmm.13840 (PMC6201366; doi:10.1111/jcmm.13840)
Supplement: Supplementary file 4 [file JCMM-22-5648-s004.doc]

**Figure S4.** Effect of NECC2 overexpression on insulin signaling.

**
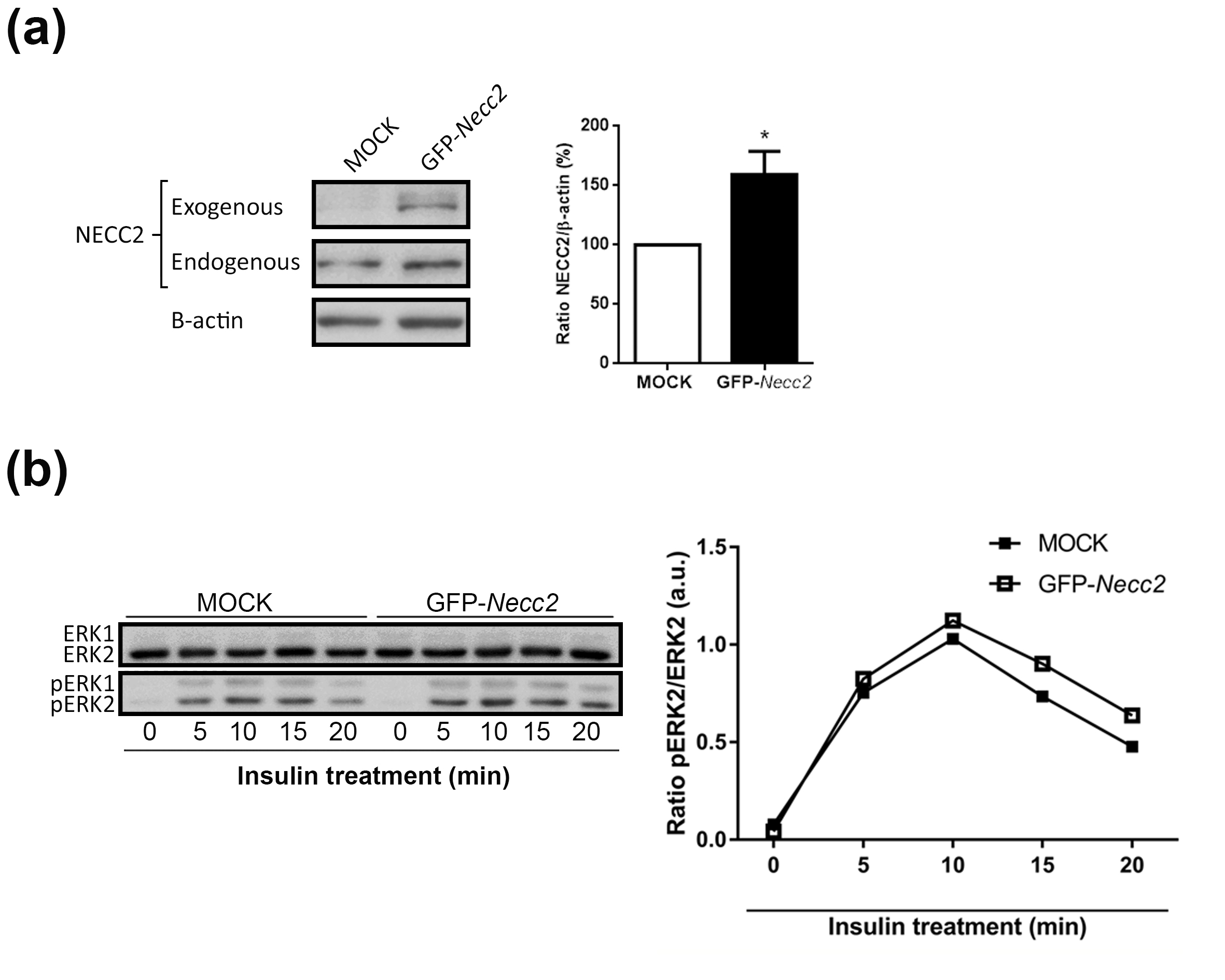
**

Immunoblot analysis of NECC2 protein content in mock-transfected and GFP-*Necc2* 3T3-L1 adipocytes using anti-NECC2 antibody (n=4) (a). Cells expressing GFP-*Necc2* or the empty vector (MOCK) were exposed for 2 h to serum-low differentiation media before 100 nmol/l insulin stimulation for the indicated time points. Whole cell protein extracts were then subjected to immunoblot with ERK2 and phospho-ERK2 (pERK2) antibodies (b).
