# Supplementary material for: The caveolae‐associated coiled‐coil protein, NECC2, regulates insulin signalling in Adipocytes
Source: J Cell Mol Med. 2018 Aug 30;22(11):5648–61. doi: 10.1111/jcmm.13840 (PMC6201366; doi:10.1111/jcmm.13840)
Supplement: Supplementary file 5 [file JCMM-22-5648-s005.doc]

**Figure S5.** Effect of NECC2 overexpression and silencing on the phosphorylation rate of ERK1.


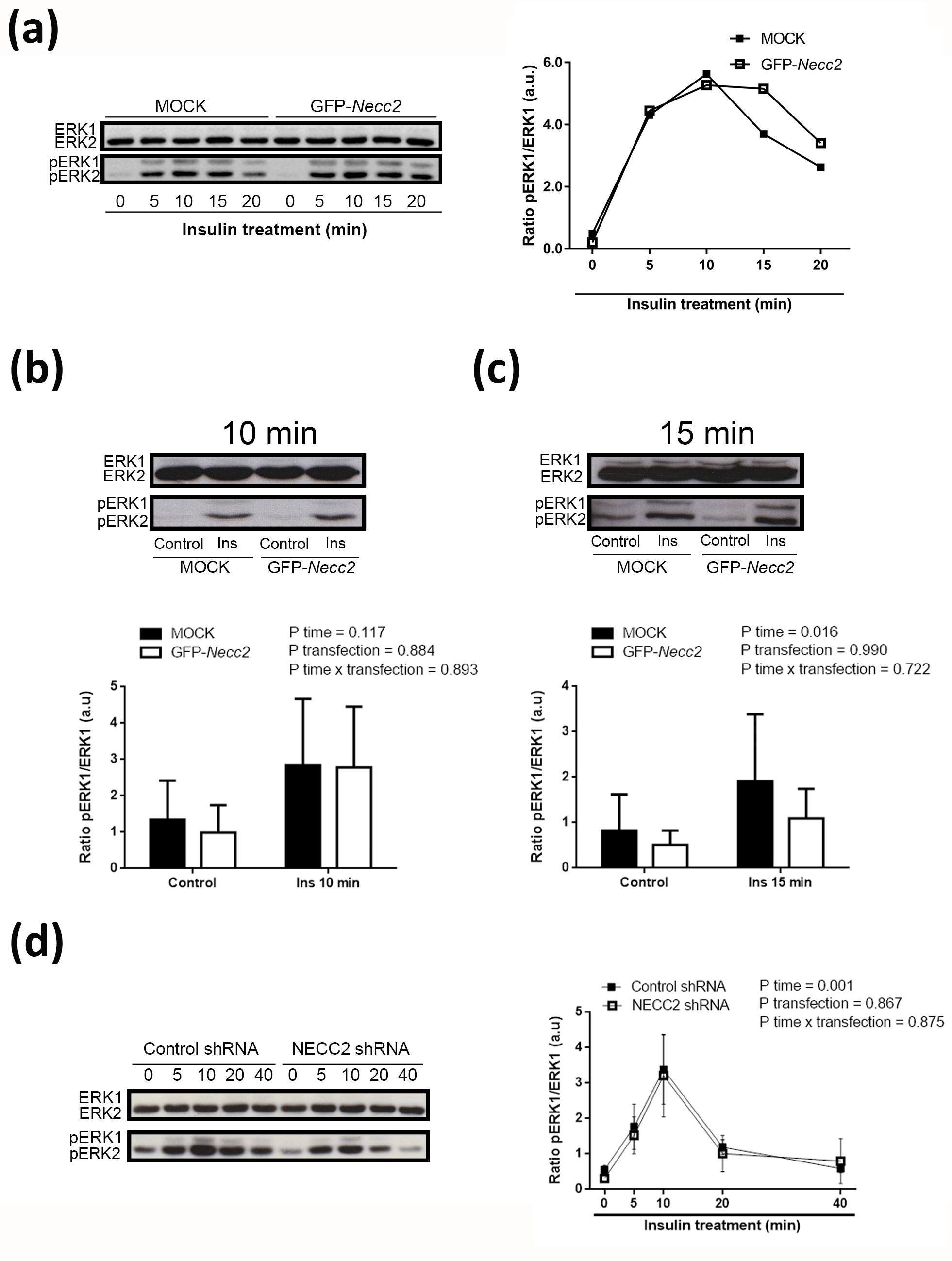


(a-c) Cells expressing GFP-*Necc2* or the empty vector (MOCK) were exposed for 2 h to serum-low differentiation media before 100 nmol/l insulin stimulation for the indicated time points. (b and c) The data represent the means ± SEM of four independent experiments. (d) 3T3-L1 adipocytes were transiently transfected with a shRNA for *Necc2* (NECC2 shRNA), or the empty vector (Control shRNA), pre-treated 2h with serum-low differentiation media and treated with insulin (100 nmol/l) during the indicated time points. The data represent the means ± SEM of four independent experiments. (a-d) Whole cell protein extracts were then subjected to immunoblot with ERK1 and phospho-ERK1 (pERK1) antibodies. Quantitative data were represented as ratio of pERK1 *vs*. ERK1. The data were analyzed using paired-samples *t* test, independent samples *t* test and RM-ANOVA was used to calculate the time effect (P time), transfection effect (P transfection) and the time x transfection interaction (P time x transfection).
