# Supplementary material for: The caveolae‐associated coiled‐coil protein, NECC2, regulates insulin signalling in Adipocytes
Source: J Cell Mol Med. 2018 Aug 30;22(11):5648–61. doi: 10.1111/jcmm.13840 (PMC6201366; doi:10.1111/jcmm.13840)
Supplement: Supplementary file 6 [file JCMM-22-5648-s006.doc]

**Figure S6.** Effect of NECC2 overexpression and silencing on caveolin-1 and insulin receptor expression.

**
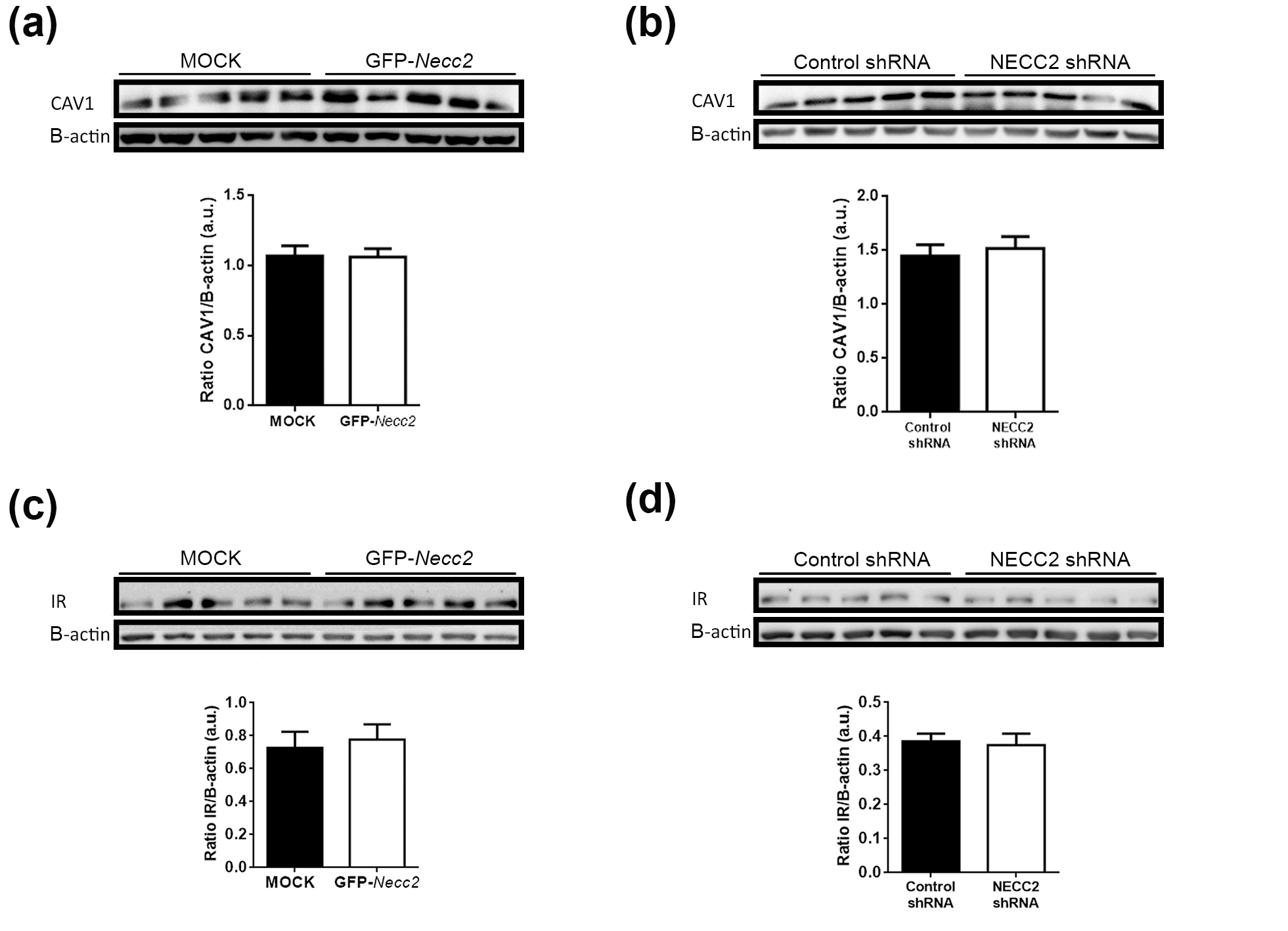
**

Differentiated 3T3-L1 cells transiently transfected with GFP-*Necc2* (a, c)*,* or NECC2 shRNA (b, d). Whole cell protein extracts were subjected to immunoblot using caveolin 1 (CAV1), insulin receptor (IR) and B-actin antibodies. The data are expressed as a ratio between target proteins and B-actin, and represent the means ± SEM (n=5). Data were analyzed using independent samples *t* test and expressed as arbitrary units (a.u.).
