# Supplementary material for: The caveolae‐associated coiled‐coil protein, NECC2, regulates insulin signalling in Adipocytes
Source: J Cell Mol Med. 2018 Aug 30;22(11):5648–61. doi: 10.1111/jcmm.13840 (PMC6201366; doi:10.1111/jcmm.13840)
Supplement: Supplementary file 7 [file JCMM-22-5648-s007.doc]

**Figure S7.** Effect of exposure of 3T3-L1 adipocytes to fatty acids on insulin response. **
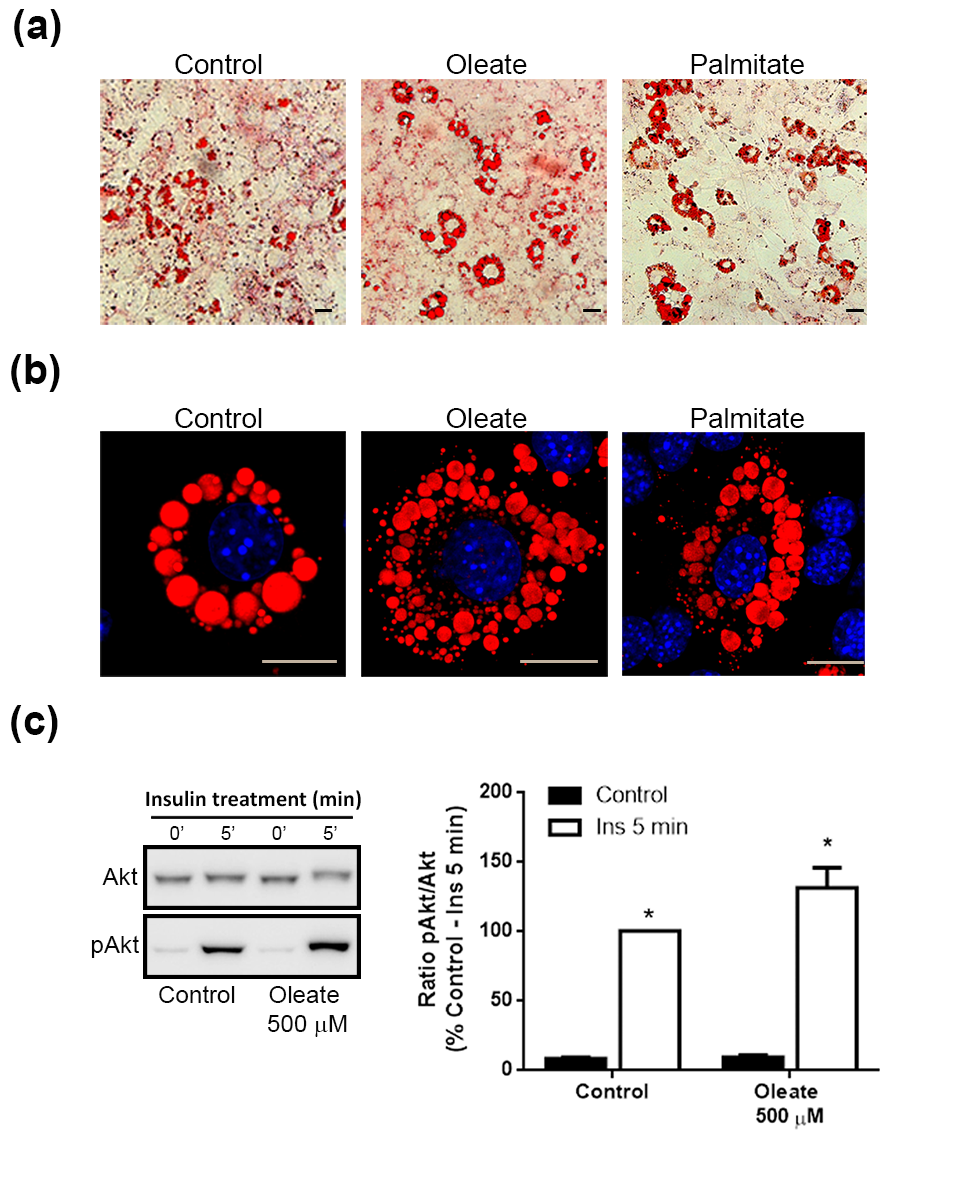
**

3T3-L1 adipocytes were exposed to oleate (500 µmol/l), or palmitate (500 µmol/l) for 18 h. Cell were then fixed and treated with Oil Red O and observed by light (n = 3) (a) and confocal microscopy (n = 2) (b). DAPI was used for nuclei labeling. Scale bar 10 μm. After exposure to oleate (500 µmol/l for 18 h), control and fatty acid-treated cells were stimulated with insulin (Ins, 100 nmol/l) for 5 min. (c). Whole cell protein extracts were then subjected to immunoblot with Akt and phospho-Akt (pAkt) antibodies. Quantitative data were represented as ratio of pAkt *vs*. Akt. The data are expressed as a percentage of values in control cultures treated with insulin (100%), and represent the means ± SEM of three independent experiments. The data were analyzed using paired-samples *t* test and independent samples *t* test. ^*^, *P* < 0.05 *vs*. corresponding control cells.
