# Supplementary material for: The caveolae‐associated coiled‐coil protein, NECC2, regulates insulin signalling in Adipocytes
Source: J Cell Mol Med. 2018 Aug 30;22(11):5648–61. doi: 10.1111/jcmm.13840 (PMC6201366; doi:10.1111/jcmm.13840)
Supplement: Supplementary file 8 [file JCMM-22-5648-s008.doc]

**Table S1.** List of antibodies employed in this study.

| Antibody | Species | Dilution | Supplier | Reference |
| --- | --- | --- | --- | --- |
|  |  |  |  |  |
| **Western blot** |  |  |  |  |
| NECC2 | rabbit | 1:1,000 | Abyntek, Spain |  |
| CAV1 | mouse | 1:1,000 | Novus Biologicals, CO | NB 100-615 |
| Cavin1 | rabbit | 1:1,000 | Abcam | AB48824 |
| IR | mouse | 1:1,000 | Santa Cruz Biotechnology, Heilderberg, Germany | SC-57342 |
| B-actin | mouse | 1:10,000 | Sigma-Aldrich, Steinheim, Germany | A2066 |
| A-Tubulin | mouse | 1:1,000 | Sigma-Aldrich | T 6199 |
| AKT | rabbit | 1:5,000 | Cell Signaling, MA | 9272 |
| p-AKT (Ser473) | rabbit | 1:2,500 | Cell Signaling | 4060 |
| ERK1/2 | rabbit | 1:5,000 | Abcam, Cambridge, UK | AB196883 |
| p-ERK1/2 (Thr202-Tyr204) | rabbit | 1:1,500 | Abcam | AB24157 |
| c-Myc | rabbit | 1:1,000 | Cell Signaling | 2278 |
| GFP | rabbit | 1:1,100 | Abcam | AB290 |
| Adiponectin | mouse | 1:500 | Chemi-Con (Billerica, EE.UU) | MAB3608 |
| **Immunocitochemistry** |  |  |  |  |
| NECC2 | rabbit | 1:500 | Abyntek, Spain |  |
| CAV1 | mouse | 1:500 | Novus Biologicals | NB 100-615 |
| IR | mouse | 1:500 | Santa Cruz Biotechnology | SC-57342 |
| Perilipin1 | guinea-pig | 1:1000 | Progen, Heidelberg, Germany | GP29 |
| Alexa594-conjugated secondary antibody | donkey | 1:500 | Invitrogen, Carlsbad, CA | A21207 |
| Alexa488-conjugated secondary antibody | chicken | 1:500 | Invitrogen | A21200 |
| Alexa488-conjugated secondary antibody | goat | 1:500 | Invitrogen | A11073 |

The references and optimized concentrations of the antibodies used in each experiment. Neuroendocrine long coiled-coil protein 2 (NECC2). Caveolin-1 (CAV1). Insulin receptor (IR). Extracellular-regulated kinase 1/2 (ERK1/2).
