# Supplementary material for: The caveolae‐associated coiled‐coil protein, NECC2, regulates insulin signalling in Adipocytes
Source: J Cell Mol Med. 2018 Aug 30;22(11):5648–61. doi: 10.1111/jcmm.13840 (PMC6201366; doi:10.1111/jcmm.13840)
Supplement: Supplementary file 9 [file JCMM-22-5648-s009.doc]

**Table S2.** Anthropometric and biochemical characteristics of subjects.

|  |  | **Obese** | | |  |
| --- | --- | --- | --- | --- | --- |
| **Variables** | **LN** | **NG** | **IGT** | **T2D** | **P** |
| n | 4 | 11 | 11 | 12 |  |
| Sex, M/F | 2/2 | 5/6 | 5/6 | 6/6 |  |
| Age, y | 41.0±2.7 | 41.3±3.7 | 42.4±1.9 | 48.7±2.5 | 0.185 |
| Body weight, kg | 66.4±7.5 | 112.5±6.4^*^ | 131.4±6.5^*^ | 125.2±7.6^*^ | **<0.001** |
|  | | | | | |
| BMI, kg/m^2^ | 22.2±0.9 | 41.2±2.0^*^ | 47.8±2.0^*^ | 44.8±2.5^*^ | **<0.001** |
| Body fat, % | 28.4 ± 2.6 | 45.9±3.3^*^ | 53.3±1.7^*^ | 47.8±2.4^*^ | **<0.001** |
| Waist circumference, cm | 73.0± 4.0 | 115.6± 3.3^*^ | 116.6± 4.5^*^ | 122.4± 4.1^*^ | **<0.001** |
|  | | | | | |
| Glucose, mmol/l | 4.9±0.2 | 5.1±0.1 | 5.8±0.2^*,†^ | 8.1±0.4^*,†,‡^ | **<0.001** |
| Insulin, pmol/l | 42.3±3.5 | 104.1±17.4^*^ | 77.0±9.7^*^ | 201.3±41.6^*^ | **0.003** |
| HOMA | 1.4±0.2 | 3.4±0.6^*^ | 3.0±0.4^*^ | 11.0±2.5^*,‡^ | **0.001** |
| Triglycerides, mmol/l | 0.8±0.1 | 1.3±0.1 | 1.4±0.5 | 1.5±0.2 | 0.661 |
| Total cholesterol, mmol/l | 5.0±0.3 | 5.1±0.3 | 5.1±0.3 | 5.4±0.4 | 0.905 |
| LDL-C, mmol/l | 3.1±0.3 | 3.3±0.3 | 3.1±0.3 | 3.5±0.3 | 0.756 |
| HDL-C, mmol/l | 1.4±0.04 | 1.0±0.1 | 1.4±0.2 | 1.0±0.1 | 0.134 |
| Uric acid, µmol/l | 267.7±23.8 | 392.6±41.6 | 398.5±29.7 | 392.6±23.8 | 0.104 |
| Fibrinogen, μmol/l | 7.3±3.5 | 10.1±0.5 | 10.3±0.8 | 10.0±1.3 | 0.682 |
| Homocysteine, μmol/l | 7.9±1.6 | 7.8±0.8 | 7.5±0.8 | 7.7±0.8 | 0.991 |
| vWF, % | 92.0±18.0 | 136.5± 15.9 | 156.7±30.2 | 161.5±17.1 | 0.591 |
| Leptin µg/l | 8.8±1.3 | 42.6±8.9^*^ | 49.5±8.5^*^ | 40.6±11.0 | **0.013** |

Data presented as mean ± SEM. Individuals were matched by age and sex in the whole cohort, and by body fat in the obese groups. BMI, body mass index; HDL, high density lipoprotein; HOMA, homeostatic model assessment; IGT, impaired glucose intolerance; LDL, low density lipoprotein; LN, lean; NG, normoglycemic; T2D, type 2 diabetes; vWF, von Willebrand factor. Differences between groups were analyzed by ANOVA followed by Tukey or Games-Howell´s test. ^*^P< 0.05 vs LN, ^†^P< 0.05 vs OB-NG, ^‡^P<0.05 vs OB-IGT. HOMA was logarithmically transformed for statistical analysis.
