# Supplementary material for: The caveolae‐associated coiled‐coil protein, NECC2, regulates insulin signalling in Adipocytes
Source: J Cell Mol Med. 2018 Aug 30;22(11):5648–61. doi: 10.1111/jcmm.13840 (PMC6201366; doi:10.1111/jcmm.13840)
Supplement: Supplementary file 10 [file JCMM-22-5648-s010.doc]

**Table S3.** Sequences of primers used for qPCR studies.

|  | Sense | Antisense |
| --- | --- | --- |
| Mouse *Necc2* | 5’-GCCATCAACTTCCACCATACA-3’ | 5’-TGGTCAGATTGGCGTTATCC-3’ |
| Human *NECC2* | 5’-GCGGGACAAGCTGTTAAGATTC-3’ | 5’-AAGCCTCTTCGTCGTATCCAAA-3’ |
| *18*s rRNA | 5´-CCCATTCGAACGTCTGCCCTATC-3´ | 5´-TGCTGCCTTCCTTGGATGTGGTA-3´ |

Specific primers designed for measure NECC2 gene expression in mouse and human cells by qPCR studies. 18s rRNA were used as an internal housekeeping gene.
